# Eco-friendly polyethylene glycol (PEG-400): a green reaction medium for one-pot, four-component synthesis of novel asymmetrical bis-spirooxindole derivatives at room temperature†

**DOI:** 10.1039/c7ra13133j

**Published:** 2018-01-09

**Authors:** Alireza Hasaninejad, Maryam Beyrati

**Affiliations:** Department of Chemistry, Faculty of Sciences, Persian Gulf University Bushehr 7516913817 Iran a_hasaninejad@yahoo.com

## Abstract

PEG-400 has been used as a green and biodegradable polymeric solvent for the one-pot, two-step, multi-component synthesis of novel asymmetrical bis-spirooxindole derivatives by the reaction of *N*-alkyl isatin, isatin derivatives, alkylmalonates and C–H activated carbonyl compounds in the presence of K_2_CO_3_ at room temperature. Using this procedure, all the products were obtained in good to excellent yields.

## Abstract 2

PEG-400 has been used for the synthesis of novel asymmetrical bis-spirooxindoles by the reaction of *N*-alkyl isatin, isatin derivatives, alkylmalonates and C–H activated carbonyl compounds in the presence of K_2_CO_3_ at room temperature.

## Graphical abstract

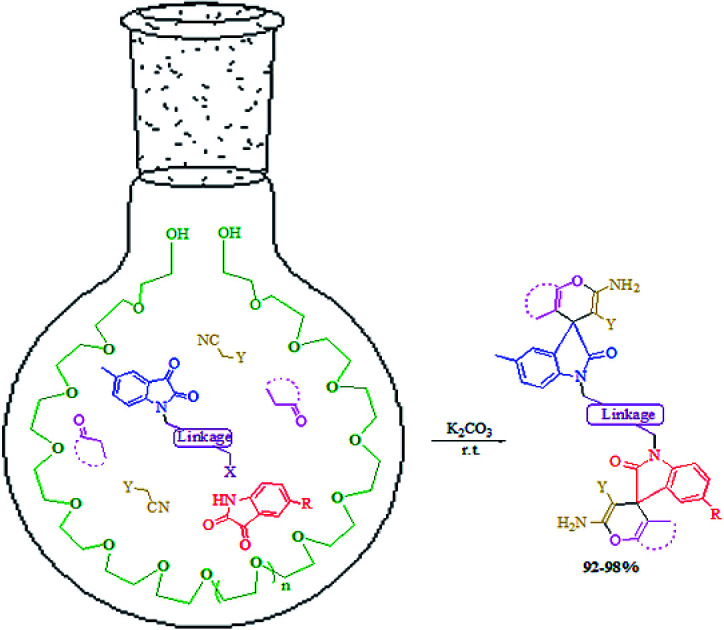

## Introduction

In recent years, the development of a high efficiency, high selectivity, green, safe, atom- and step-economical synthesis strategy has become of paramount importance in the field of organic chemistry. One-pot, multi-component reactions are a powerful tool in organic and medicinal chemistry because they can shorten the reaction time, simplify the separation step, reduce costs, and give a relatively higher total chemical yield compared to multistep synthesis.^1,2^

Solvents are widely used in organic synthesis and have been a cause of major concern due to their associated environmental hazards. Therefore, replacement of harmful organic solvents with an eco-friendly medium is one of the major focal points of green chemistry.^3–10^ Polyethylene glycol (PEG) and modified polyethylene glycol derivatives have become more popular alternate reaction media, due to their interesting properties like non-toxicity, bio-compatibility, and bio-degradability. Moreover, PEG is considered as a natural, inexpensive, safe, recyclable, degradable, non-flammable, facile, environmentally benign and abundantly available green solvent.^11^

**Scheme 1:**
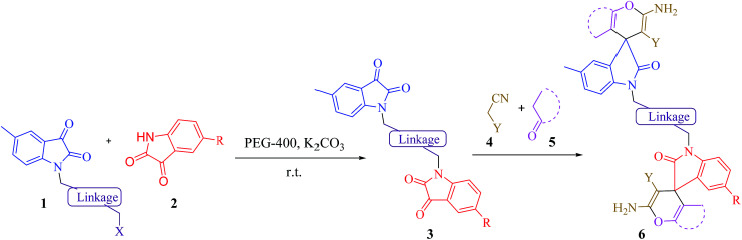
The synthesis of novel asymmetrical bis-spirooxindoles 6*via* the reaction between *N*-alkyl isatin 1, isatin derivatives 2, alkyl malonates 4 and carbonyl compounds 5 in the presence of K_2_CO_3_in PEG-400 at room temperature.

Spirooxindoles are commonly occurring heterocyclic ring systems and are found in many natural products and pharmaceuticals.^12,13^ These compounds are known to display anti-tubercular,^14^ antifungal,^15^ anti-mycobacterial,^16^ anti-tumor,^17,18^ anti-malarial^19^ and anti-microbial^20^ activities. Until recently, there have been few reports on the synthesis of symmetric bis-spirooxindole derivatives.^21–24^ In continuation of our research interest in the synthesis of biologically important heterocyclic compounds,^25–33^ herein we report a synthetic route for the preparation of novel asymmetrical bis-spirooxindoles 6*via* a one-pot four-component condensation reaction of *N*-alkyl isatin 1 (1 eq.), isatin derivatives 2 (1 eq.), alkyl malonates 4 (2 eq.) and C–H activated carbonyl compounds 5 (2 eq.) in the presence of K_2_CO_3_ in polyethylene glycol medium at room temperature (Scheme 1).

## Results and discussion

**Scheme 2:**
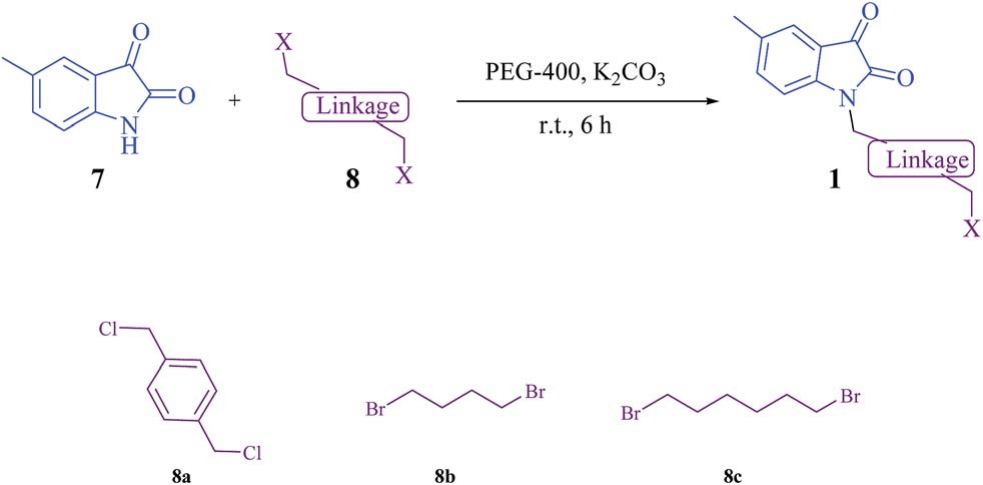
Synthetic pathway for the synthesis of *N*-alkyl isatin derivatives.

At first, the *N*-alkyl isatins 1 were prepared from the reaction of 5-methyl isatin (7, 1 eq.) with dihalidederivatives (8, excess) in the presence of K_2_CO_3_ in PEG-400 at room temperature (Scheme 2).

Then, to optimize the reaction conditions for the synthesis of asymmetrical bis spirooxindole derivatives, the reaction of 1-(4-(chloromethyl)benzyl)-5-methylindoline-2,3-dione (1a), isatin (2a), malononitrile (4a) and 1,3-cyclohexanedione (5b) its behavior was studied in the presence of different catalytic systems, and the results are summarized in Table 1. As it is shown in Table 1, higher yield and shorter reaction time were obtained when the reaction was carried out in the presence of 1 mmol of K_2_CO_3_ in PEG-400 at room temperature (Table 1, entry 9).

**Table 1:** Effects of reagent and solvent on the reaction of 1-(4-(chloromethyl)benzyl)-5-methylindoline-2,3-dione, isatin, malononitrile and 1,3-cyclohexanedion under different conditions^a^

Entry	Reagent	reagent (mmol)	Solvent	Temp. (°C)	Time (h)	Yield (%)
1	—	—	PEG-400	r.t.	48	—
2	l-Proline	1.0	PEG-400	r.t.	48	—
3	DABCO	1.0	PEG-400	r.t.	48	—
4	Et_3_N	1.0	PEG-400	r.t.	48	—
5	Cs_2_CO_3_	1.0	PEG-400	r.t.	24	65
6	Na_2_CO_3_	1.0	PEG-400	r.t.	24	30
7	NaHCO_3_	1.0	PEG-400	r.t.	24	—
8	CaCO_3_	1.0	PEG-400	r.t.	24	25
9	K_2_CO_3_	1.0	PEG-400	r.t.	7	95
10	K_2_CO_3_	0.5	PEG-400	r.t.	15	70
11	K_2_CO_3_	0.8	PEG-400	r.t.	15	87
12	K_2_CO_3_	1.5	PEG-400	r.t.	7	94
13	K_2_CO_3_	1.0	MeCN	r.t.	48	Trace
14	K_2_CO_3_	1.0	H_2_O	r.t.	48	—
15	K_2_CO_3_	1.0	EtOH	r.t.	48	—
16	K_2_CO_3_	1.0	MeOH	r.t.	24	—
17	K_2_CO_3_	1.0	*n*-Propanol	r.t.	24	—
18	K_2_CO_3_	1.0	*tert*-Butanol	r.t.	24	—
19	K_2_CO_3_	1.0	DMSO	r.t.	13	80
20	K_2_CO_3_	1.0	DMF	r.t.	15	80
21	K_2_CO_3_	1.0	—	80	48	—
22	K_2_CO_3_	1.0	—	100	48	—

a Isolated yields.

**Fig. 1:**
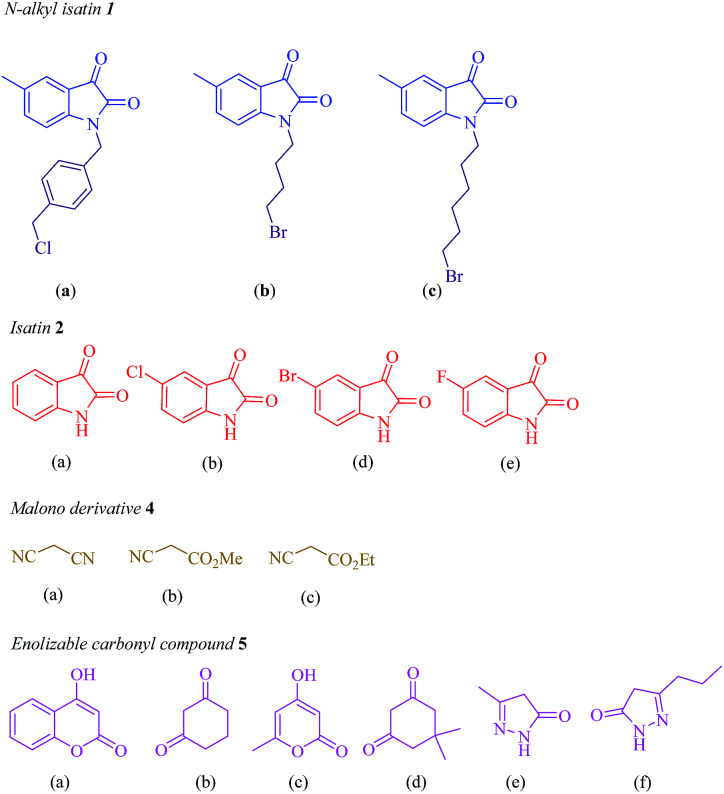
Diversity elements employed for synthesis of asymmetrical bis-spirooxindoles.

Subsequently, the scope and efficiency of the reagent were explored under the optimized reaction conditions for the condensation of different asymmetrical bis-isatins 3 with a broad range of structurally diverse carbonyl compounds and alkyl malonatesto furnish the related products. The structural diversity of reactants is summarized in Fig. 1 and the results are displayed in Table 2.

**Table 2:** One-pot, four-component synthesis of asymmetrical bis-spirooxindole derivatives in the presence of K_2_CO_3_ in PEG-400 at room temperature^a^

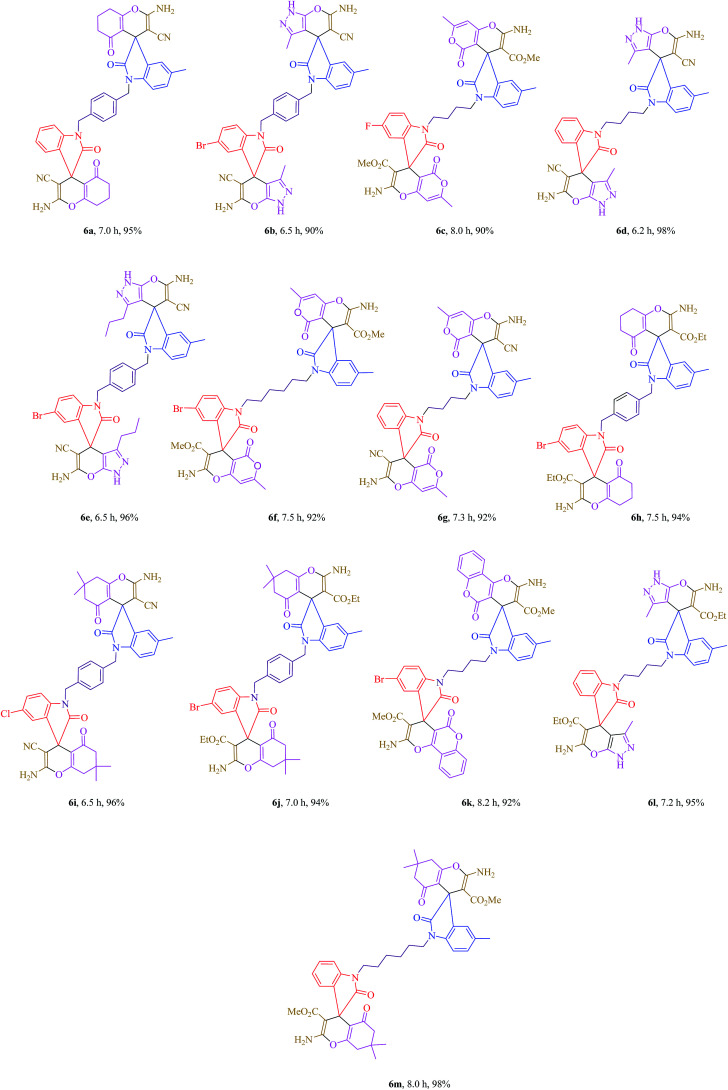

a Isolated yields.

The synthetic pathway for the synthesis of titled compounds is consisting of two steps. At first, asymmetrical bis-isatin derivatives are obtained from the condensation reaction of *N*-alkyl isatin 1 and isatin derivatives 2. Then, the resulting products are treated with alkyl malonates 4 and carbonyl compounds 5 to afford the related asymmetrical bis-spirooxindole derivatives as the desired products.

As Table 2 indicates, a variety of isatin derivatives, alkyl malonates and carbonyl compounds were successfully applied in this process to afford the corresponding asymmetrical bis-spirooxindole derivatives as novel and potentially biologically important compounds in excellent yields.

## Experimental

All chemicals were purchased from Merck or Fluka chemical companies. The ^1^H NMR (400 MHz) and ^13^C NMR (100 MHz) were run on a BrukerAvance400. Melting points were recorded on a Stuart Scientific Apparatus SMP3 (UK) in open capillary tubes. Elemental C, H and N analyses were performed using a Costech CHNS–O elemental analyzer.

### General procedure for the synthesis of asymmetrical bisspirooxindolederivatives 6

K_2_CO_3_ (1 mmol) was added to a stirred mixture of *N*-alkylisatin 1 (1 mmol), isatin derivative 2 (1 mmol) in PEG-400 (3 mL) and the reaction mixture was stirred at room temperature to complete the formation of related asymmetrical bis-isatin 3 (monitored by TLC). Subsequently, alkyl malonates 4 (2 mmol) and cyclic ketone 5 (2 mmol) were added to this reaction mixture and reacted at room temperature for the appropriate amount of time (see Table 2). After completion of the reaction, 3 ml of water was added to the reaction mixture; the precipitate was filtrated and recrystallized from hot ethanol to afford the pure products.

#### 2-Amino-1′-(4-((2-amino-3-cyano-5′-methyl-2′,5-dioxo-5,6,7,8-tetrahydrospiro[chromene-4,3′-indolin]-1′-yl)methyl)benzyl)-2′,5-dioxo-5,6,7,8-tetrahydrospiro[chromene-4,3′-indoline]-3-carbonitrile 6a

White powder, mp > 270 °C, ^1^H NMR (DMSO-*d*_6_,400 MHz) *δ* (ppm): 1.96 (t, *J* = 6.0 Hz, 4H), 2.23 (s, 3H), 2.25–2.31 (m, 4H), 2.70–2.73 (m, 4H), 4.88 (d, *J* = 10.0 Hz, 4H), 6.56 (d, *J* = 8.4 Hz, 1H), 6.69 (d, *J* = 7.6 Hz, 1H), 6.93–6.99 (m, 3H), 7.12–7.16 (m, 3H), 7.34 (d, *J* = 8.8 Hz, 3H), 7.45 (s, 4H). ^13^C NMR (DMSO-*d*_6_, 100 MHz) *δ* (ppm): 20.2, 21.1, 27.3, 31.2, 36.8, 43.6, 47.1, 47.2, 57.7, 57.9, 70.3, 109.1, 109.3, 112.2, 112.2, 117.9, 117.9, 123.0, 123.6, 124.2, 127.6, 128.7, 128.9, 131.9, 134.2, 134.2, 135.5, 135.6, 140.7, 143.0, 159.1, 159.2, 159.2, 166.8, 166.9, 177.2, 177.3, 195.6. Anal. calcd for C_43_H_34_N_6_O_6_: C, 70.67; H, 4.69; N, 11.50%. Found: C, 70.65; H, 4.72; N, 11.48%.

#### 6′-Amino-1-(4-((6′-amino-5′-cyano-3′,5-dimethyl-2-oxo-1′*H*-spiro[indoline-3,4′-pyrano[2,3-*c*]pyrazol]-1-yl)methyl)benzyl)-5-bromo-3′-methyl-2-oxo-1′*H*-spiro[indoline-3,4′-pyrano[2,3-*c*]pyrazole]-5′-carbonitrile 6b

Cream powder, mp > 270 °C, ^1^H NMR (DMSO-*d*_6_,400 MHz) *δ* (ppm): 0.98 (s, 3H), 1.05 (s, 3H), 2.35 (s, 3H), 4.71 (d, *J* = 15.6 Hz, 2H), 4.97 (d, *J* = 15.6 Hz, 2H), 6.65–6.69 (m, 2H), 6.85–7.06 (m, 6H), 7.42 (s, 1H), 7.59 (s, 4H), 7.97 (s, 1H), 12.43 (s, 2H). ^13^C NMR (DMSO-*d*_6_, 100 MHz) *δ* (ppm): 21.1, 27.7, 28.0, 32.5, 43.6, 47.1, 50.4, 57.8, 109.2, 128.3, 130.3, 132.5, 133.3, 138.1, 139.0, 139.2, 140.1, 140.2, 142.0, 143.0, 146.3, 147.6, 148.3, 149.7, 149.9, 156.5, 157.1, 159.5, 171.2, 173.0, 173.8. Anal. calcd for C_39_H_29_BrN_10_O_4_: C, 59.93; H, 3.74; Br, 10.22; N, 17.92%. Found: C, 59.96; H, 3.72; N, 17.90%.

#### Methyl 2′-amino-1-(4-(2′-amino-3′-(methoxycarbonyl)-5,7′-dimethyl-2,5′-dioxo-5′*H*-spiro[indoline-3,4′-pyrano[4,3-*b*]pyran]-1-yl)butyl)-5-fluoro-7′-methyl-2,5′-dioxo-5′*H*-spiro[indoline-3,4′-pyrano[4,3-*b*]pyran]-3′-carboxylate 6c

Cream powder, mp > 270 °C, ^1^H NMR (DMSO-*d*_6_,400 MHz) *δ* (ppm): 1.49–1.51 (m, 2H), 1.64–1.72 (m, 2H), 2.21 (s, 3H), 2.98 (s, 3H), 3.02 (s, 3H), 3.23 (s, 3H), 3.37 (s, 3H), 3.55–3.64 (m, 2H), 3.71–3.74 (m, 2H), 6.32 (s, 2H), 6.80–6.93 (m, 2H), 6.99–7.00 (m, 1H), 7.11–7.19 (m, 2H), 8.03 (s, 2H), 8.08 (s, 2H). ^13^C NMR (DMSO-*d*_6_, 100 MHz) *δ* (ppm): 27.5, 27.7, 28.0, 32.5, 43.5, 47.2, 50.3, 56.9, 110.0, 110.1, 117.7, 121.0, 121.5, 125.5, 127.7, 128.4, 129.3, 130.6, 137.9, 157.6, 159.6, 163.1, 163.9, 165.6, 167.9, 178.6, 195.7. Anal. calcd for C_41_H_35_FN_4_O_12_: C, 61.96; H, 4.44; F, 2.39; N, 7.05%. Found: C, 61.94; H, 4.42; N, 7.08%.

#### 6′-Amino-1-(4-(6′-amino-5′-cyano-3′,5-dimethyl-2-oxo-1′*H*-spiro[indoline-3,4′-pyrano[2,3-*c*]pyrazol]-1-yl)butyl)-3′-methyl-2-oxo-1′*H*-spiro[indoline-3,4′-pyrano[2,3-*c*]pyrazole]-5′-carbonitrile 6d

Pink powder, mp > 270 °C, ^1^H NMR (DMSO-*d*_6_,400 MHz) *δ* (ppm): 1.31–1.42 (m, 2H), 1.57–1.65 (m, 2H), 2.05 (s, 3H), 3.08 (s, 6H), 3.60–3.68 (m, 4H), 6.90–6.94 (m, 1H), 6.99 (d, *J* = 8.4 Hz, 2H), 7.42 (dd, *J* = 8.4, 2.0 Hz, 4H), 7.49–7.52 (m, 2H), 8.06 (s, 4H), 12.03 (s, 2H). ^13^C NMR (DMSO-*d*_6_, 100 MHz) *δ* (ppm): 14.1, 14.4, 25.0, 44.5, 47.3, 59.3, 62.4, 121.2, 123.4, 125.4, 125.7, 127.0, 127.7, 127.9, 128.3, 130.3, 134.5, 134.7, 135.6, 139.3, 147.4, 152.6, 155.4, 157.5, 158.5, 159.08, 159.3. Anal. calcd for C_35_H_30_N_10_O_4_: C, 64.21; H, 4.62; N, 21.39%. Found: C, 64.22; H, 4.60; N, 21.41%.

#### 6′-Amino-1-(4-((6′-amino-5′-cyano-5-methyl-2-oxo-3′-propyl-1′*H*-spiro[indoline-3,4′-pyrano[2,3-*c*]pyrazol]-1-yl)methyl)benzyl)-5-bromo-2-oxo-3′-propyl-1′*H*-spiro[indoline-3,4′-pyrano[2,3-*c*]pyrazole]-5′-carbonitrile 6e

White powder, mp > 270 °C, ^1^H NMR (DMSO-*d*_6_, 400 MHz) *δ* (ppm): 0.86–0.92 (m, 3H), 1.02–1.08 (m, 3H), 1.91–1.94 (m, 2H), 2.19–2.25 (m, 2H), 2.69 (s, 3H), 3.01–3.06 (m, 4H), 4.67–4.79 (m, 2H), 4.88–4.99 (m, 2H), 6.65–6.68 (m, 3H), 6.82–6.85 (m, 2H), 6.94 (d, *J* = 6.8 Hz, 1H), 7.02–7.06 (m, 4H), 7.59 (s, 2H), 7.91 (s, 2H), 12.21 (s, 2H). ^13^C NMR (DMSO-*d*_6_, 100 MHz) *δ* (ppm): 14.1, 14.4, 25.0, 27.1, 28.3, 32.1, 45.0, 46.6, 51.1, 59.0, 62.4, 76.6, 113.9, 114.0, 119.6, 119.7, 124.9, 125.6, 126.1, 129.6, 130.7, 130.9, 133.9, 136.2, 136.2, 137.5, 137.6, 142.6, 144.9, 161.1, 161.2, 161.2, 168.8, 168.9, 179.2, 179.3. Anal. calcd for C_43_H_37_BrN_10_O_4_: C, 61.65; H, 4.45; Br, 9.54; N, 16.72%. Found: C, 61.67; H, 4.43; N, 16.71%.

#### Methyl 2′-amino-1-(6-(2′-amino-3′-(methoxycarbonyl)-5,7′-dimethyl-2,5′-dioxo-5′*H*-spiro[indoline-3,4′-pyrano[4,3-*b*]pyran]-1-yl)hexyl)-5-bromo-7′-methyl-2,5′-dioxo-5′*H*-spiro[indoline-3,4′-pyrano[4,3-*b*]pyran]-3′-carboxylate 6f

White powder, mp > 270 °C, ^1^H NMR (DMSO-*d*_6_, 400 MHz) *δ* (ppm): 1.02–1.08 (m, 2H), 1.46–1.49 (m, 2H), 1.59–1.63 (m, 4H), 2.10 (s, 3H), 2.20 (s, 6H), 3.36 (s, 6H), 3.59–3.72 (m, 2H), 6.31 (s, 2H), 6.79 (d, *J* = 8.4 Hz, 3H), 6.97 (d, *J* = 7.2 Hz, 2H), 7.60 (d, *J* = 7.6 Hz, 1H), 8.02 (s, 4H). ^13^C NMR (DMSO-*d*_6_, 100 MHz) *δ* (ppm): 14.0, 19.6, 21.0, 26.7, 27.4, 46.5, 50.9, 59.3, 76.0, 114.2, 125.0, 128.1, 129.3, 129.6, 130.4, 133.3, 135.4, 137.7, 137.8, 138.9, 139.9, 140.2, 142.5, 146.4, 146.7, 147.4, 149.1, 149.4, 159.5, 164.7, 167.4, 170.3, 171.1, 171.1, 174.6. Anal. calcd for C_43_H_39_BrN_4_O_12_: C, 58.44; H, 4.45; Br, 9.04; N, 6.34%. Found: C, 58.42; H, 4.44; N, 6.36%.

#### 2′-Amino-1-(4-(2′-amino-3′-cyano-5,7′-dimethyl-2,5′-dioxo-5′*H*-spiro[indoline-3,4′-pyrano[4,3-*b*]pyran]-1-yl)butyl)-7′-methyl-2,5′-dioxo-5′*H*-spiro[indoline-3,4′-pyrano[4,3-*b*]pyran]-3′-carbonitrile 6g

Cream powder, mp > 270 °C, ^1^H NMR (DMSO-*d*_6_, 400 MHz) *δ* (ppm): 1.03–1.08 (m, 4H), 2.10 (s, 3H), 2.23 (s, 3H), 2.24 (s, 3H), 3.59–3.65 (m, 2H), 3.74–3.80 (m, 2H), 6.34 (s, 2H), 6.86–6.91 (m, 1H), 6.97–7.02 (m, 4H), 7.14–7.20 (m, 2H), 8.09 (s, 4H). ^13^C NMR (DMSO-*d*_6_, 100 MHz) *δ* (ppm): 14.2, 27.2, 28.2, 32.1, 44.4, 46.7, 51.0, 59.1, 76.7, 113.3, 114.3, 114.5, 118.3, 120.2, 122.4, 122.7, 124.2, 124.8, 124.9, 125.3, 127.3, 131.1, 131.7, 132.6, 136.3, 144.3, 149.6, 152.4, 155.5, 155.5, 156.0, 156.3, 169.3. Anal. calcd for C_39_H_30_N_6_O_8_: C, 65.91; H, 4.25; N, 11.83%. Found: C, 65.89; H, 4.26; N, 11.85%.

#### Ethyl 2-amino-1′-(4-((2-amino-3-(ethoxycarbonyl)-5′-methyl-2′,5-dioxo-5,6,7,8-tetrahydrospiro[chromene-4,3′-indolin]-1′-yl)methyl)benzyl)-5′-bromo-2′,5-dioxo-5,6,7,8-tetrahydrospiro[chromene-4,3′-indoline]-3-carboxylate 6h

Pink powder, mp > 270 °C, ^1^H NMR (DMSO-*d*_6_, 400 MHz) *δ* (ppm): 0.38 (q, *J* = 7.2 Hz, 3H), 0.59 (q, *J* = 7.2 Hz, 3H), 1.92 (t, *J* = 6.0 Hz, 4H), 2.10 (s, 3H), 2.20–2.30 (m, 4H), 2.69 (t, *J* = 5.8 Hz, 4H), 3.41–3.49 (m, 2H), 3.76–3.83 (m, 2H), 4.69 (d, *J* = 16.0 Hz, 2H), 4.97 (dd, *J* = 15.8, 2.6 Hz, 2H), 6.64 (dd, *J* = 8.0, 2.4 Hz, 4H), 7.17 (d, *J* = 1.6 Hz, 1H), 7.25 (d, *J* = 8.4 Hz, 4H), 7.56 (s, 4H), 8.02 (s, 1H). Sample solubility was too low for ^13^C-NMR even after heating. Anal. calcd for C_47_H_43_BrN_4_O_10_: C, 62.46; H, 4.80; Br, 8.84; N, 6.20%. Found: C, 62.47; H, 4.78; N, 6.22%.

#### 2-Amino-1′-(4-((2-amino-3-cyano-5′,7,7-trimethyl-2′,5-dioxo-5,6,7,8-tetrahydrospiro[chromene-4,3′-indolin]-1′-yl)methyl)benzyl)-5′-chloro-7,7-dimethyl-2′,5-dioxo-5,6,7,8-tetrahydrospiro[chromene-4,3′-indoline]-3-carbonitrile 6i

Cream powder, mp > 270 °C, ^1^H NMR (DMSO-*d*_6_, 400 MHz) *δ* (ppm): 0.57 (d, *J* = 2.4 Hz, 1H), 0.59 (d, *J* = 2.4 Hz, 1H), 0.99 (s, 3H), 1.04 (s, 3H), 1.23 (s, 3H), 1.25 (s, 3H), 2.90 (s, 2H), 4.66 (d, *J* = 16.0 Hz, 2H), 4.96 (d, *J* = 15.6 Hz, 2H), 6.52 (d, *J* = 7.6 Hz, 3H), 6.75 (s, 1H), 6.85 (d, *J* = 7.6 Hz, 4H), 7.57 (s, 4H), 7.95 (s, 1H). ^13^C NMR (DMSO-*d*_6_, 100 MHz) *δ* (ppm): 10.4, 11.3, 20.2, 21.2, 27.3, 31.2, 36.7, 43.5, 47.1, 47.2, 57.7, 57.9, 70.3, 109.1, 109.3, 112.2, 112.3, 117.9, 117.9, 123.0, 123.6, 124.2, 127.5, 128.8, 128.9, 132.0, 134.2, 134.2, 135.4, 135.6, 140.6, 143.0, 159.2, 159.2, 159.2, 166.8, 166.9, 177.2, 177.3, 192.5, 192.6. Anal. calcd for C_47_H_41_ClN_6_O_6_: C, 68.73; H, 5.03; Cl, 4.32; N, 10.23%. Found: C, 68.74; H, 5.05; N, 10.21%.

#### Ethyl 2-amino-1′-(4-((2-amino-3-(ethoxycarbonyl)-5′,7,7-trimethyl-2′,5-dioxo-5,6,7,8-tetrahydrospiro[chromene-4,3′-indolin]-1′-yl)methyl)benzyl)-5′-bromo-7,7-dimethyl-2′,5-dioxo-5,6,7,8-tetrahydrospiro[chromene-4,3′-indoline]-3-carboxylate 6j

White powder, mp > 270 °C, ^1^H NMR (DMSO-*d*_6_, 400 MHz) *δ* (ppm): 0.55 (t, *J* = 6.4 Hz, 6H), 0.98 (s, 6H), 1.05 (s, 6H), 2.04–2.11 (m, 2H), 2.21 (d, *J* = 16.0 Hz, 2H), 2.62–2.69 (m, 2H), 2.91 (s, 3H), 3.05 (d, *J* = 9.6 Hz, 2H), 3.72–3.78 (m, 4H), 4.71 (d, *J* = 16.4 Hz, 1H), 4.76 (d, *J* = 16.0 Hz, 1H), 4.90 (d, *J* = 15.6 Hz, 1H), 4.97 (d, *J* = 15.6 Hz, 1H), 6.68 (d, *J* = 3.6 Hz, 2H), 6.86 (d, *J* = 7.2.0 Hz, 4H), 6.94 (d, *J* = 6.0 Hz, 2H), 7.05 (s, 1H), 7.59 (s, 4H), 7.95 (d, *J* = 4.4 Hz, 1H). ^13^C NMR (DMSO-*d*_6_, 100 MHz) *δ* (ppm): 13.9, 21.0, 26.2, 27.1, 31.2, 42.8, 46.8, 50.9, 51.1, 53.4, 62.4, 62.5, 76.9, 79.1, 122.9, 123.5, 124.1, 127.5, 128.7, 128.9, 132.0, 134.2, 134.2, 135.4, 135.6, 140.7, 143.0, 159.1, 159.2, 159.2, 166.8, 166.9, 177.2, 177.3, 195.7. Anal. calcd for C_51_H_51_BrN_4_O_10_: C, 63.82; H, 5.36; Br, 8.32; N, 5.84%. Found: C, 63.81; H, 5.38; N, 5.82%.

#### Methyl 2′-amino-1-(4-(2′-amino-3′-(methoxycarbonyl)-5-methyl-2,5′-dioxo-5′*H*-spiro[indoline-3,4′-pyrano[3,2-*c*]chromen]-1-yl)butyl)-5-bromo-2,5′-dioxo-5′*H*-spiro[indoline-3,4′-pyrano[3,2-*c*]chromene]-3′-carboxylate 6k

White powder, mp > 270 °C, ^1^H NMR (DMSO-*d*_6_, 400 MHz) *δ* (ppm): 0.67–0.74 (m, 4H), 1.85 (s, 3H), 3.04 (s, 3H), 3.27 (s, 3H), 3.66–3.72 (m, 2H), 3.75–3.82 (m, 2H), 6.86–6.89 (m, 2H), 7.04–7.08 (m, 3H), 7.10 (d, *J* = 7.2 Hz, 2H), 7.19 (t, *J* = 7.0 Hz, 1H), 7.47 (d, *J* = 6.8 Hz, 1H), 7.55 (t, *J* = 7.6 Hz, 2H), 7.77 (t, *J* = 7.8 Hz, 2H), 8.07 (d, *J* = 8.0 Hz, 1H), 8.21 (s, 4H). ^13^C NMR (DMSO-*d*_6_, 100 MHz) *δ* (ppm): 14.1, 20.1, 27.4, 44.3, 46.8, 50.9, 53.7, 76.7, 113.2, 125.7, 128.0, 129.3, 129.5, 130.6, 133.3, 135.4, 137.7, 137.7, 139.0, 140.9, 141.2, 143.7, 147.2, 147.8, 148.4, 149.1, 149.4, 159.4, 164.6, 167.4, 169.5, 170.1, 170.1, 173.4. Anal. calcd for C_47_H_35_BrN_4_O_12_: C, 60.85; H, 3.80; Br, 8.61; N, 6.04%. Found: C, 60.87; H, 3.82; N, 6.01%.

#### Ethyl 6′-amino-1-(4-(6′-amino-5′-(ethoxycarbonyl)-3′,5-dimethyl-2-oxo-1′*H*-spiro[indoline-3,4′-pyrano[2,3-*c*]pyrazol]-1-yl)butyl)-3′-methyl-2-oxo-1′*H*-spiro[indoline-3,4′-pyrano[2,3-*c*]pyrazole]-5′-carboxylate 6l

Cream powder, mp > 270 °C, ^1^H NMR (DMSO-*d*_6_, 400 MHz) *δ* (ppm): 0.64–0.70 (m, 6H), 0.96 (s, 6H), 1.83 (s, 3H), 2.02–2.06 (m, 2H), 2.59–2.61 (m, 2H), 3.34–3.48 (m, 4H), 3.58–3.64 (m, 2H), 3.72–3.78 (m, 2H), 6.85 (td, *J* = 7.4, 2.4 Hz, 2H), 6.91–6.95 (m, 3H), 7.11 (d, *J* = 8.0 Hz, 1H), 7.14 (d, *J* = 7.6 Hz, 1H), 7.94 (s, 4H), 12.43 (s, 2H). ^13^C NMR (DMSO-*d*_6_, 100 MHz) *δ* (ppm): 14.2, 27.1, 27.2, 28.2, 28.3, 31.2, 32.1, 44.3, 45.2, 46.7, 50.9, 59.1, 76.7, 101.6, 121.3, 124.2, 126.2, 130.7, 131.9, 133.0, 133.0, 135.7, 140.0, 140.4, 141.0, 141.3, 142.8, 152.5, 161.4, 164.2, 167.5, 167.5. Anal. calcd for C_39_H_40_N_8_O_8_: C, 62.56; H, 5.38; N, 14.96%. Found: C, 62.57; H, 5.40; N, 14.94%.

#### Methyl 2-amino-1′-(6-(2-amino-3-(methoxycarbonyl)-5′,7,7-trimethyl-2′,5-dioxo-5,6,7,8-tetrahydrospiro[chromene-4,3′-indolin]-1′-yl)hexyl)-7,7-dimethyl-2′,5-dioxo-5,6,7,8-tetrahydrospiro[chromene-4,3′-indoline]-3-carboxylate 6m

White powder, mp > 270 °C, ^1^H NMR (DMSO-*d*_6_, 400 MHz) *δ* (ppm): 0.70 (s, 3H), 0.93 (s, 6H), 1.01 (s, 6H), 1.45–1.52 (m, 2H), 1.61–1.69 (m, 2H), 1.98 (d, *J* = 15.6 Hz, 2H), 2.11 (d, *J* = 15.6 Hz, 2H), 2.15 (d, *J* = 16.0 Hz, 4H), 2.91 (s, 6H), 3.10–3.21 (m, 4H), 3.62 (t, *J* = 7.0 Hz, 4H), 6.82–6.91 (m, 3H), 7.13 (t, *J* = 7.6 Hz, 2H), 7.21 (d, *J* = 8.0 Hz, 2H), 7.88 (s, 4H). ^13^C NMR (DMSO-*d*_6_, 100 MHz) *δ* (ppm): 14.4, 24.8, 25.0, 27.1, 28.3, 32.1, 42.8, 44.6, 46.8, 50.9, 51.1, 53.4, 62.4, 123.0, 126.5, 128.6, 129.2, 134.3, 135.0, 135.4, 136.1, 136.3, 137.6, 138.9, 142.3, 143.3, 144.5, 145.6, 145.9, 152.5, 153.1, 155.5, 167.2, 169.2, 169.9, 202.5. Anal. calcd for C_47_H_52_N_4_O_10_: C, 67.77; H, 6.29; N, 6.73%. Found: C, 67.75; H, 6.31; N, 6.72%.

## Conclusions

In conclusion, we have reported a highly efficient method for the synthesis of asymmetrical bis-spirooxindole derivatives *via* a one-pot, two-step, four-component condensation reaction using K_2_CO_3_ in PEG-400 as a green and biodegradable polymeric solvent at room temperature.

## Conflicts of interest

There are no conflicts to declare.

## Supplementary Material

RA-008-C7RA13133J-s001
