# Supplementary material for: Eco-friendly polyethylene glycol (PEG-400): a green reaction medium for one-pot, four-component synthesis of novel asymmetrical bis-spirooxindole derivatives at room temperature
Source: RSC Adv. 2018 Jan 9;8(4):1934–9. doi: 10.1039/c7ra13133j (PMC9077237; doi:10.1039/c7ra13133j)
Supplement: RA-008-C7RA13133J-s001 [file RA-008-C7RA13133J-s001.pdf]

Electronic Supplementary Material (ESI) for RSC Advances.
This journal is © The Royal Society of Chemistry 2018

Eco-friendly polyethylene glycol (PEG-400): a green reaction medium for one-pot, four-component synthesis of novel asymmetrical bis-spirooxindole derivatives at room temperature

Alireza Hasaninejad,†Maryam Beyrati†*

2-amino-1'-(4-((2-amino-3-cyano-5'-methyl-2',5-dioxo-5,6,7,8-tetrahydrospiro[chromene-4,3'-indolin]-1'-yl)methyl)benzyl)-2',5-dioxo-5,6,7,8-tetrahydrospiro[chromene-4,3'-indoline]-3-carbonitrile **6a**

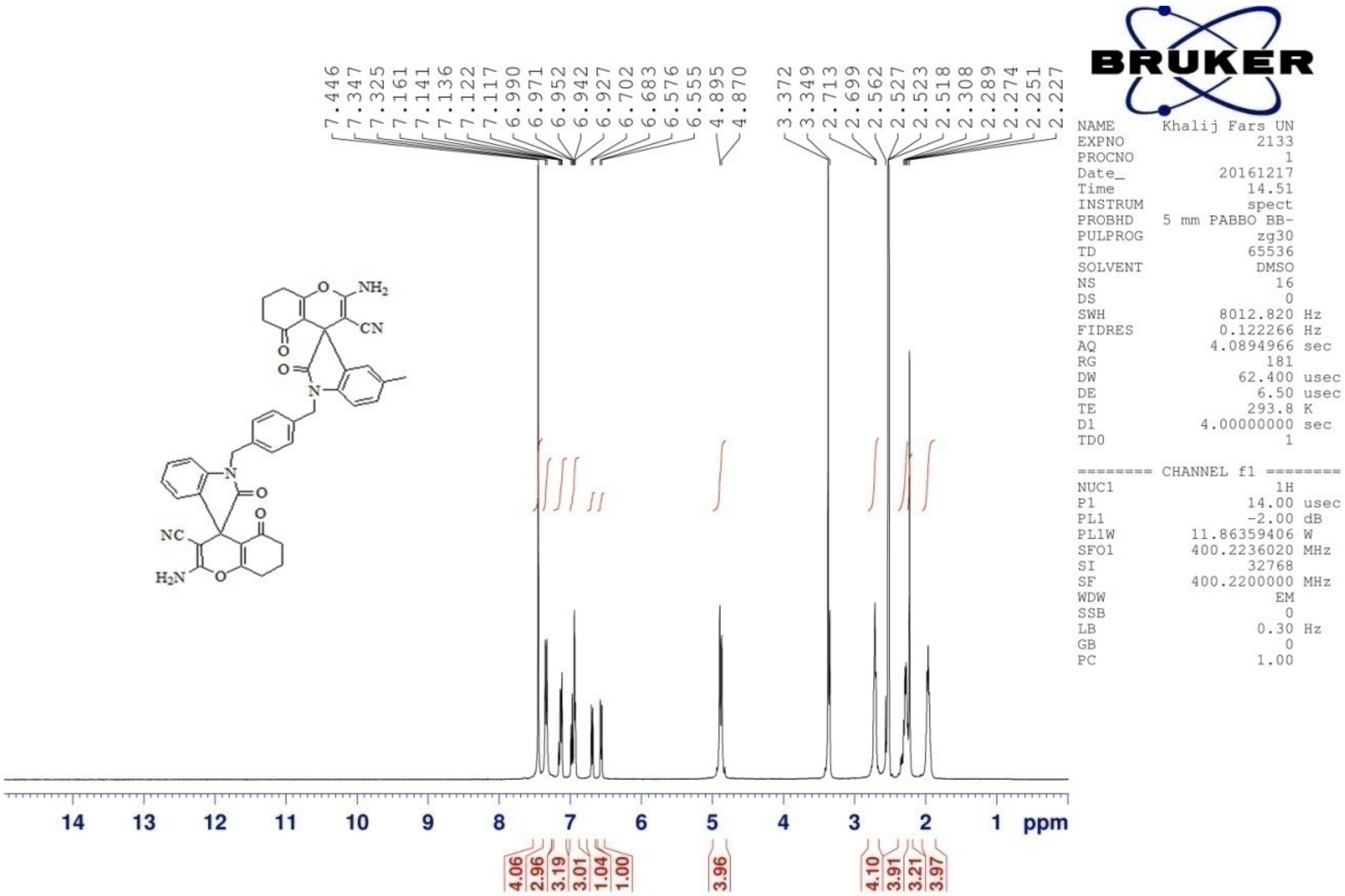

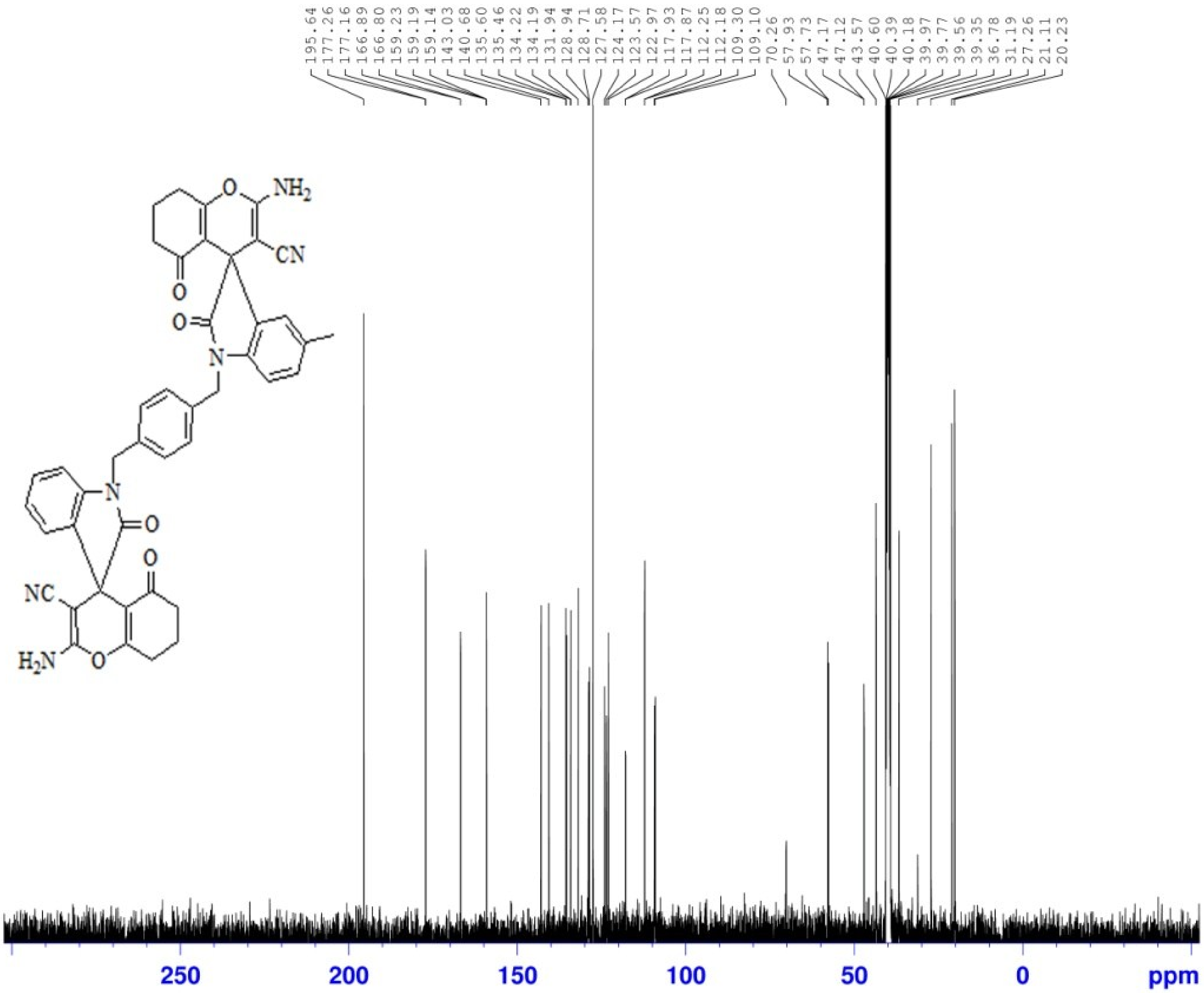

NAME	Khalij Fars UN	
EXPNO	2170	
PROCNO	1	
Date_	20170103	
Time	10.15	
INSTRUM	spect	
PROBHD	5 mm PABBO BB-	
PULPROG	zgpg30	
TD	65536	
SOLVENT	DMSO	
NS	2510	
DS	0	
SWH	35714.285	Hz
FIDRES	0.544957	Hz
AQ	0.9175540	sec
RG	2050	
DW	14.000	usec
DE	6.50	usec
TE	294.4	K
D1	1.00000000	sec
D11	0.03000000	sec
TD0	1	
======== CHANNEL f1 ========		
NUC1	13C	
P1	9.00	usec
PL1	-0.90	dB
PL1W	42.02801895	W
SFO1	100.6479784	MHz
======== CHANNEL f2 ========		
CPDPRG2	waltz16	
NUC2	1H	
PCPD2	90.00	usec
PL2	-2.00	dB
PL12	14.16	dB
PL13	17.90	dB
PL2W	11.86359406	W
PL12W	0.28722104	W
PL13W	0.12139934	W
SFO2	400.2216009	MHz
SI	32768	
SF	100.6353990	MHz
WDW	EM	
SSB	0	
LB	1.00	Hz
GB	0	
PC	1.40	

6'-amino-1-(4-((6'-amino-5'-cyano-3',5-dimethyl-2-oxo-1'H-spiro[indoline-3,4'-pyrano[2,3-c]pyrazol]-1-yl)methyl)benzyl)-5-bromo-3'-methyl-2-oxo-1'H-spiro[indoline-3,4'-pyrano[2,3-c]pyrazole]-5'-carbonitrile **6b**

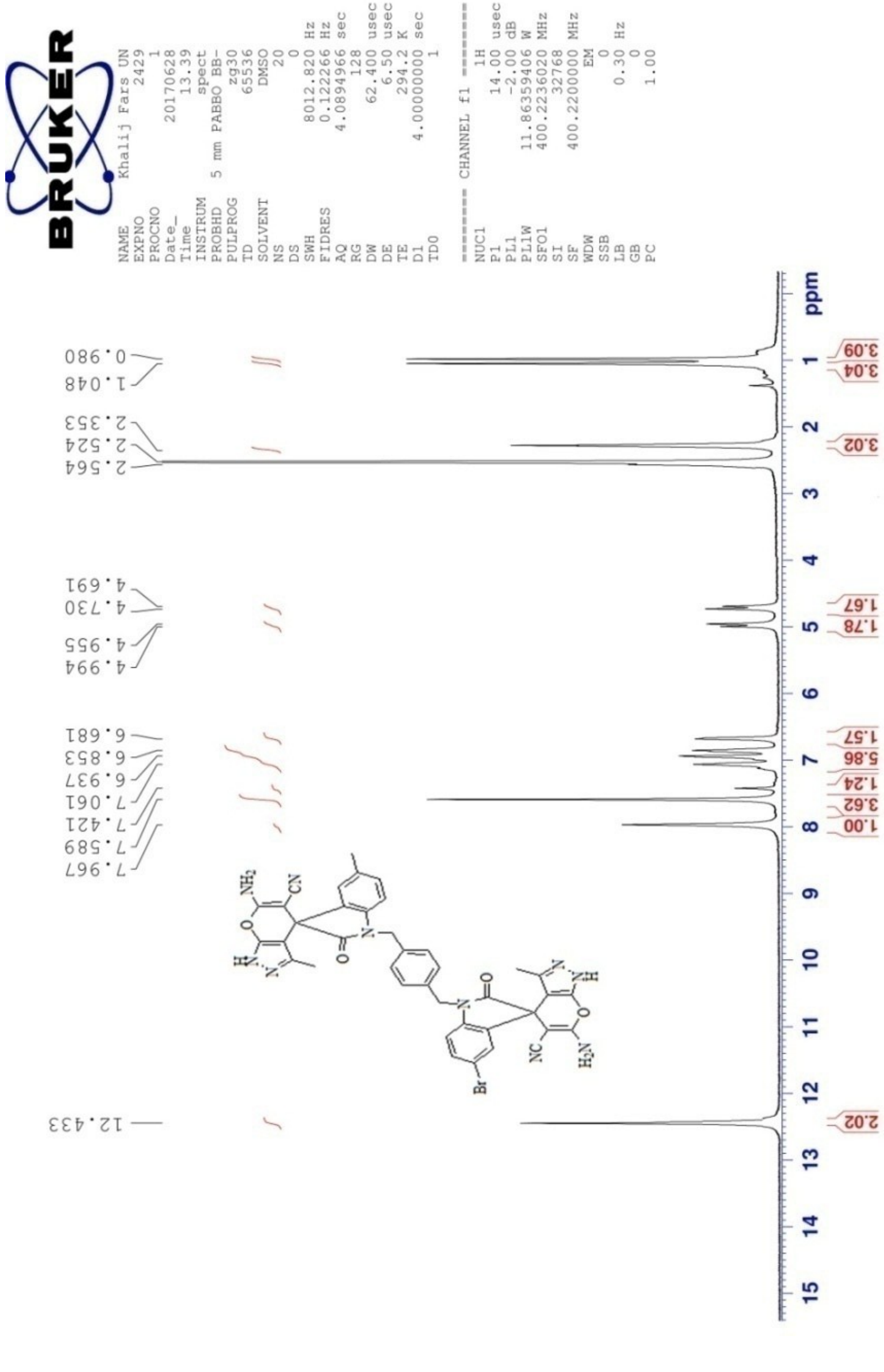

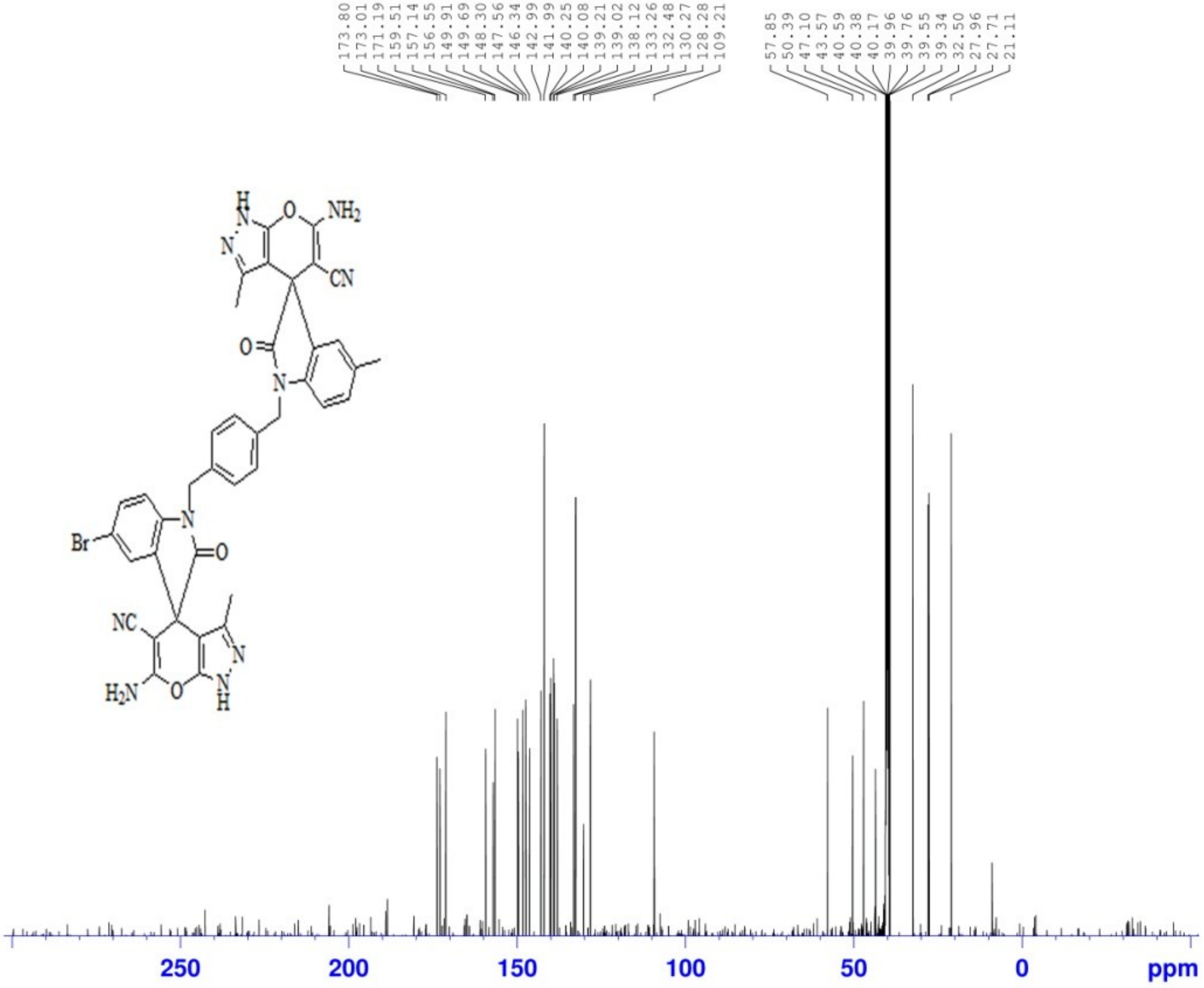

BRUKER

NAME	Khalij Fars UN
EXPNO	2450
PROCNO	1
Date_	20170719
Time	8.11
INSTRUM	spect
PROBHD	5 mm PABBO BB-
PULPROG	zgpg30
TD	65536
SOLVENT	DMSO
NS	600
DS	0
SWH	35714.285 Hz
FIDRES	0.544957 Hz
AQ	0.9175540 sec
RG	2050
DW	14.000 usec
DE	6.50 usec
TE	292.8 K
D1	1.00000000 sec
D11	0.03000000 sec
TD0	1
======== CHANNEL f1 ========	
NUC1	13C
P1	9.00 usec
PL1	-0.90 dB
PL1W	42.02801895 W
SFO1	100.6479784 MHz
======== CHANNEL f2 ========	
CPDPRG2	waltz16
NUC2	1H
PCPD2	90.00 usec
PL2	-2.00 dB
PL12	14.48 dB
PL13	17.90 dB
PL2W	11.86359406 W
PL12W	0.26681873 W
PL13W	0.12139934 W
SFO2	400.2216009 MHz
SI	32768
SF	100.6353990 MHz
WDW	EM
SSB	0
LB	1.00 Hz
GB	0
PC	1.40

methyl 2'-amino-1-(4-(2'-amino-3'-(methoxycarbonyl)-5,7'-dimethyl-2,5'-dioxo-5'H-spiro[indoline-3,4'-pyrano[4,3-b]pyran]-1-yl)butyl)-5-fluoro-7'-methyl-2,5'-dioxo-5'H-spiro[indoline-3,4'-pyrano[4,3-b]pyran]-3'-carboxylate **6c**

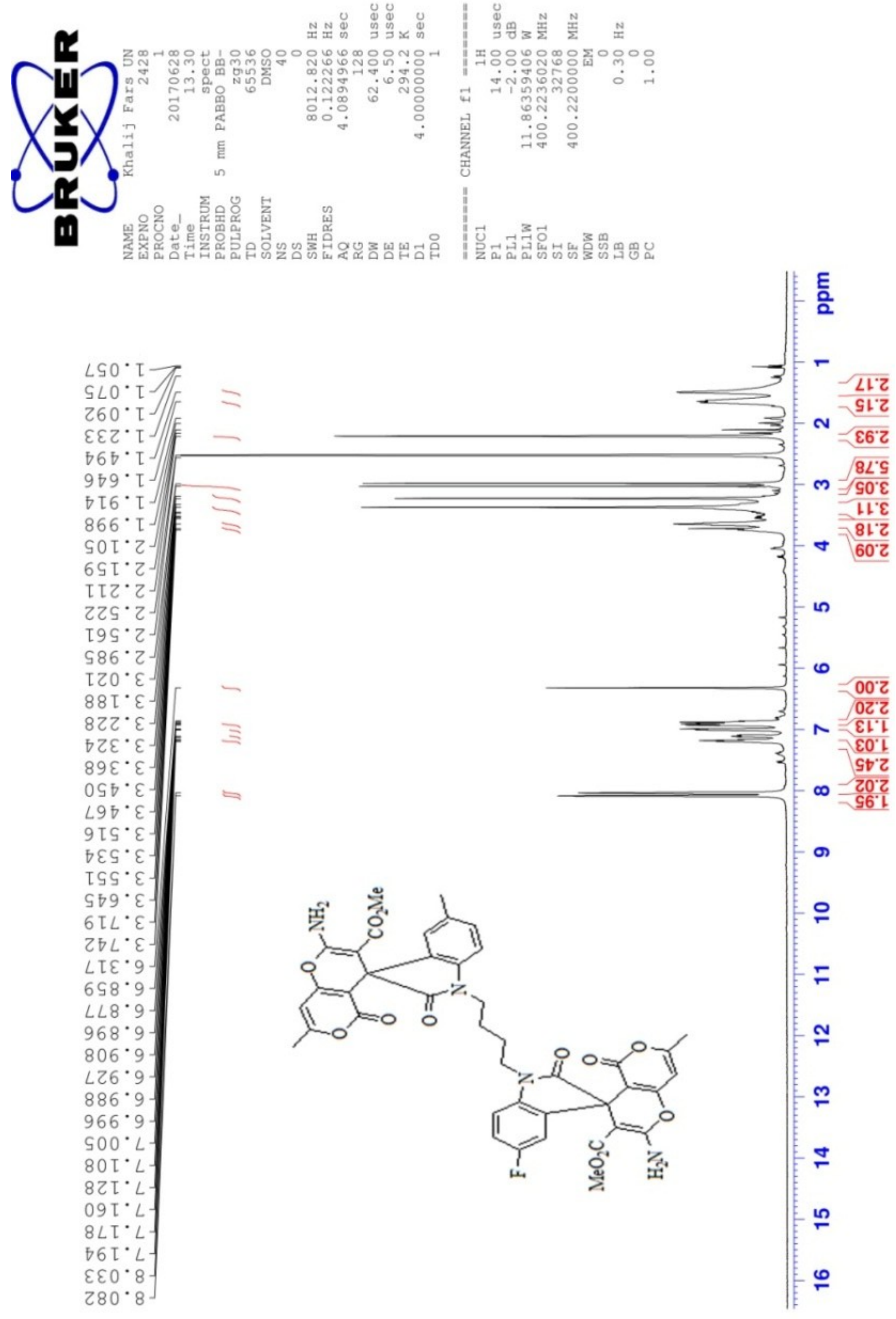

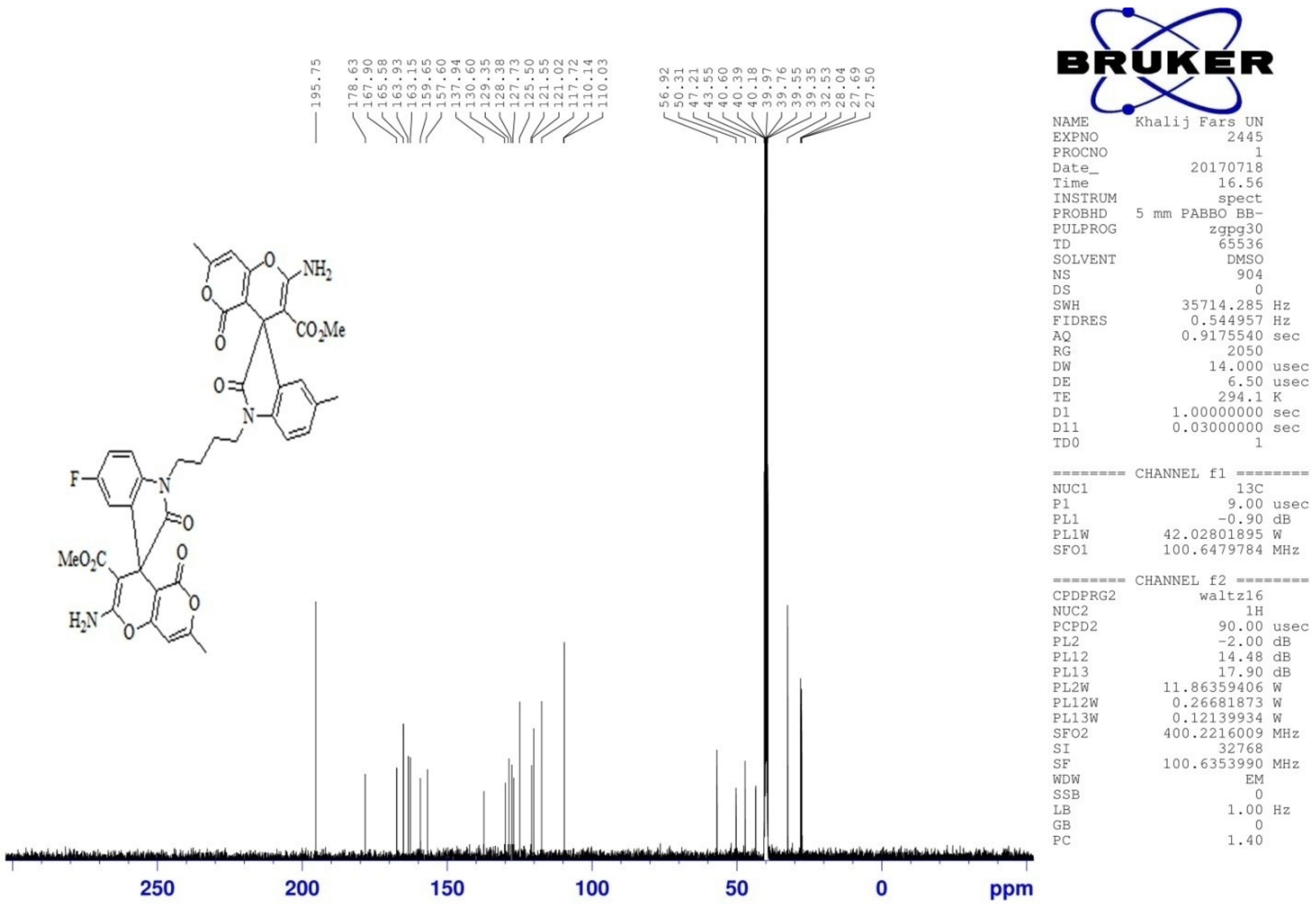
195.75
178.63
167.90
165.58
163.93
163.15
159.65
157.60
137.94
130.60
129.35
128.38
127.73
125.50
121.55
121.02
117.72
110.14
110.03
56.92
50.31
47.21
43.55
40.60
40.39
40.18
39.97
39.76
39.55
39.35
32.53
28.04
27.69
27.50
NH2
CO2Me
O
N
F
MeO2C
H2N
250
200
150
100
50
0
ppm
BRUKER
NAME Khalij Fars UN
EXPNO 2445
PROCNO 1
Date_ 20170718
Time 16.56
INSTRUM spect
PROBHD 5 mm PABBO BB-
PULPROG zgpg30
TD 65536
SOLVENT DMSO
NS 904
DS 0
SWH 35714.285 Hz
FIDRES 0.544957 Hz
AQ 0.9175540 sec
RG 2050
DW 14.000 usec
DE 6.50 usec
TE 294.1 K
D1 1.00000000 sec
D11 0.03000000 sec
TD0 1
======== CHANNEL f1 ========
NUC1 13C
P1 9.00 usec
PL1 -0.90 dB
PL1W 42.02801895 W
SFO1 100.6479784 MHz
======== CHANNEL f2 ========
CPDPRG2 waltz16
NUC2 1H
PCPD2 90.00 usec
PL2 -2.00 dB
PL12 14.48 dB
PL13 17.90 dB
PL2W 11.86359406 W
PL12W 0.26681873 W
PL13W 0.12139934 W
SFO2 400.2216009 MHz
SI 32768
SF 100.6353990 MHz
WDW EM
SSB 0
LB 1.00 Hz
GB 0
PC 1.40

6'-amino-1-(4-(6'-amino-5'-cyano-3',5-dimethyl-2-oxo-1'H-spiro[indoline-3,4'-pyrano[2,3-c]pyrazol]-1-yl)butyl)-3'-methyl-2-oxo-1'H-spiro[indoline-3,4'-pyrano[2,3-c]pyrazole]-5'-carbonitrile **6d**

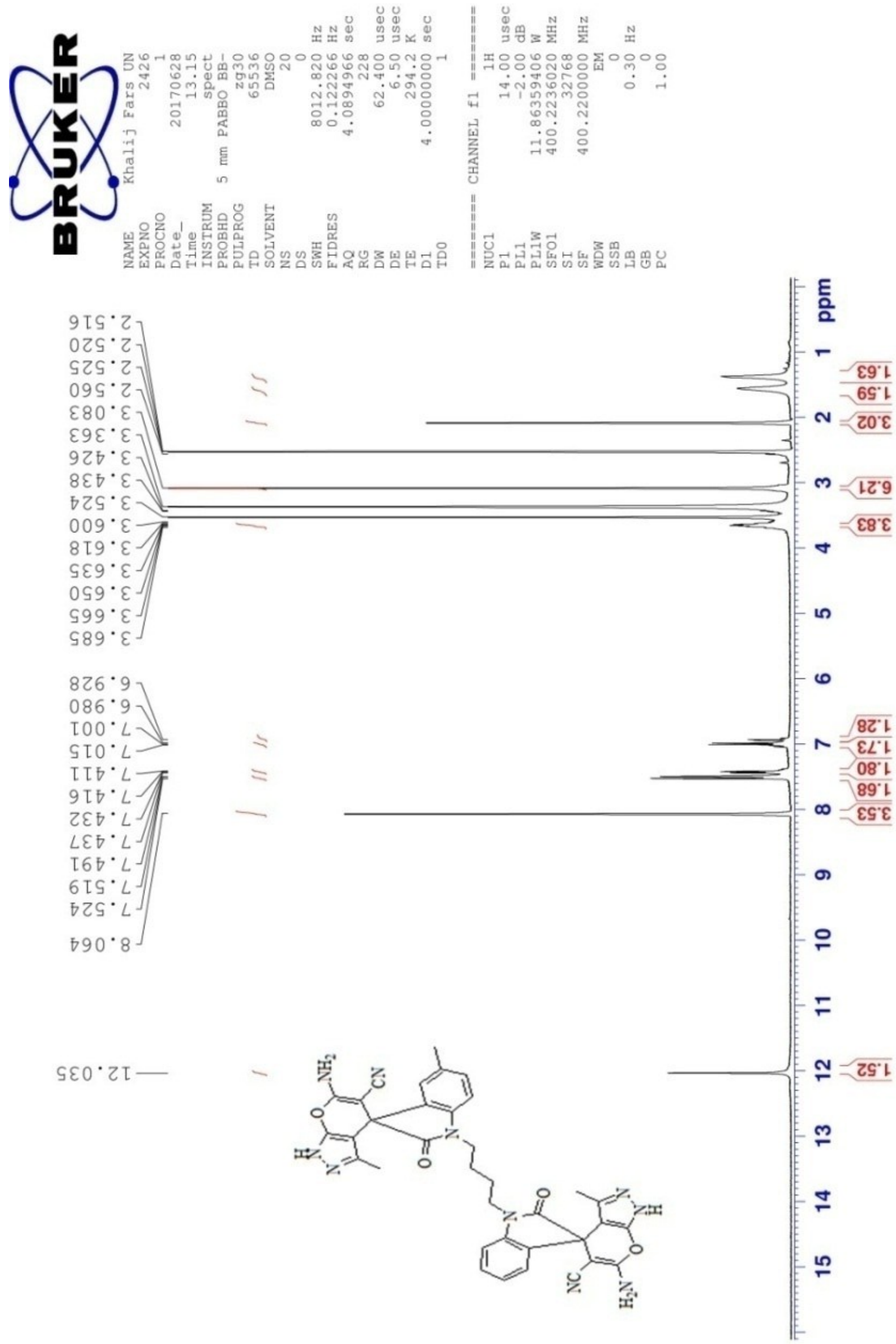

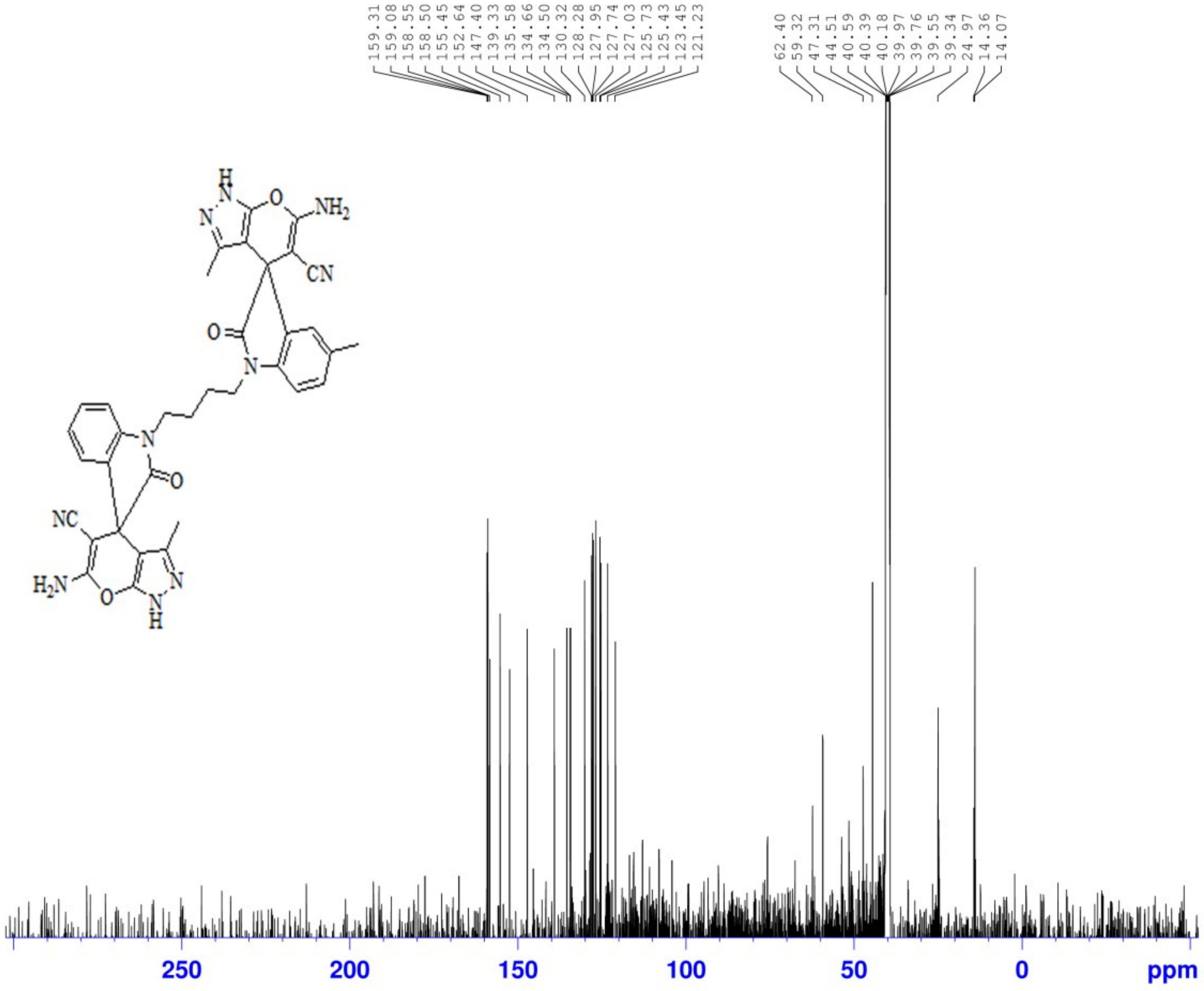

NAME	Khalij Fars UN	
EXPNO	2456	
PROCNO	1	
Date_	20170719	
Time	17.56	
INSTRUM	spect	
PROBHD	5 mm PABBO BB-	
PULPROG	zgpg30	
TD	65536	
SOLVENT	DMSO	
NS	6330	
DS	0	
SWH	35714.285	Hz
FIDRES	0.544957	Hz
AQ	0.9175540	sec
RG	2050	
DW	14.000	usec
DE	6.50	usec
TE	293.4	K
D1	1.00000000	sec
D11	0.03000000	sec
TD0	1	
======== CHANNEL f1 ========		
NUC1	13C	
P1	9.00	usec
PL1	-0.90	dB
PL1W	42.02801895	W
SFO1	100.6479784	MHz
======== CHANNEL f2 ========		
CPDPRG2	waltz16	
NUC2	1H	
PCPD2	90.00	usec
PL2	-2.00	dB
PL12	14.48	dB
PL13	17.90	dB
PL2W	11.86359406	W
PL12W	0.26681873	W
PL13W	0.12139934	W
SFO2	400.2216009	MHz
SI	32768	
SF	100.6353990	MHz
WDW	EM	
SSB	0	
LB	1.00	Hz
GB	0	
PC	1.40	

6'-amino-1-(4-((6'-amino-5'-cyano-5-methyl-2-oxo-3'-propyl-1'H-spiro[indoline-3,4'-pyrano[2,3-c]pyrazol]-1-yl)methyl)benzyl)-5-bromo-2-oxo-3'-propyl-1'H-spiro[indoline-3,4'-pyrano[2,3-c]pyrazole]-5'-carbonitrile **6e**

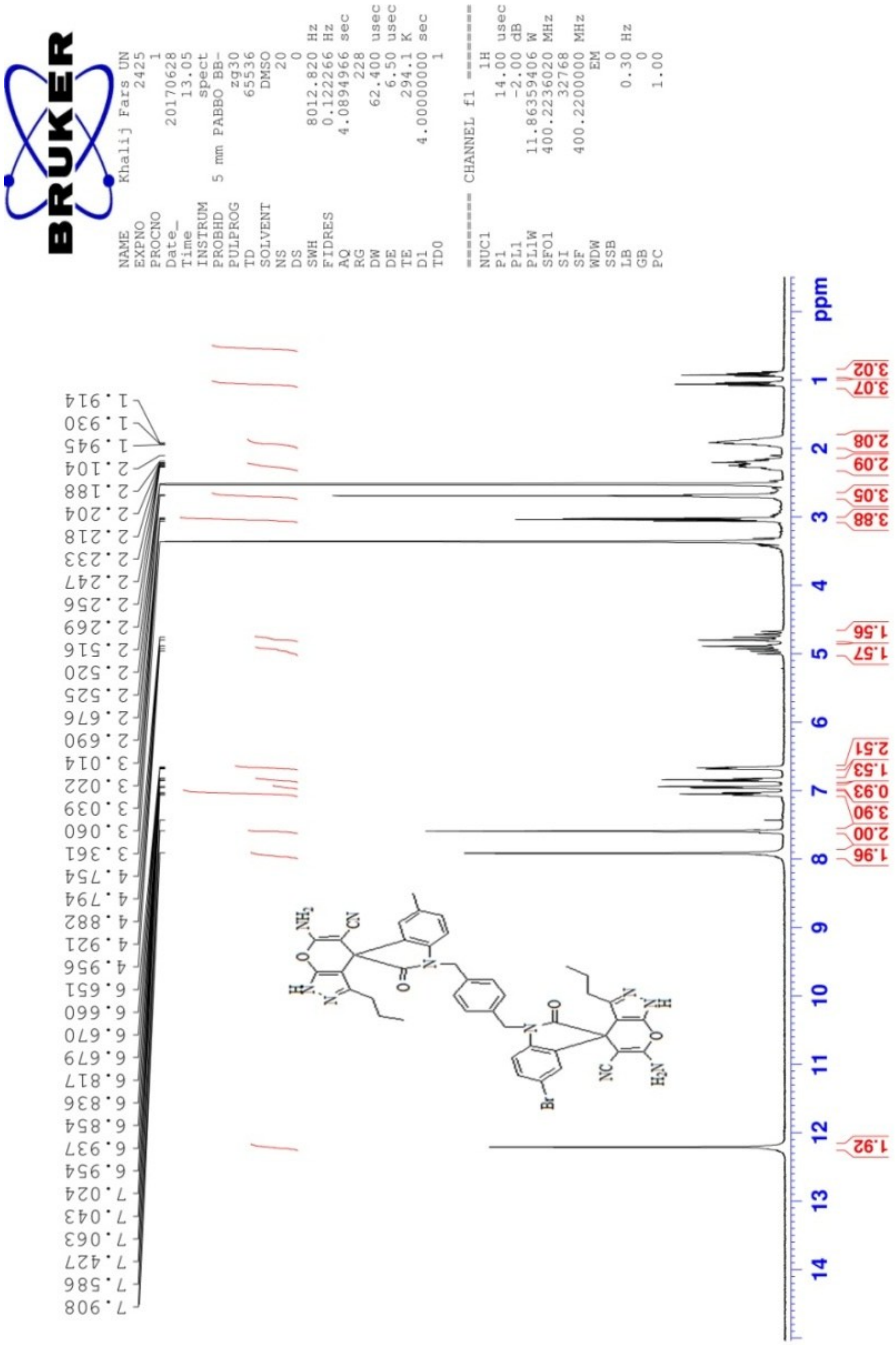

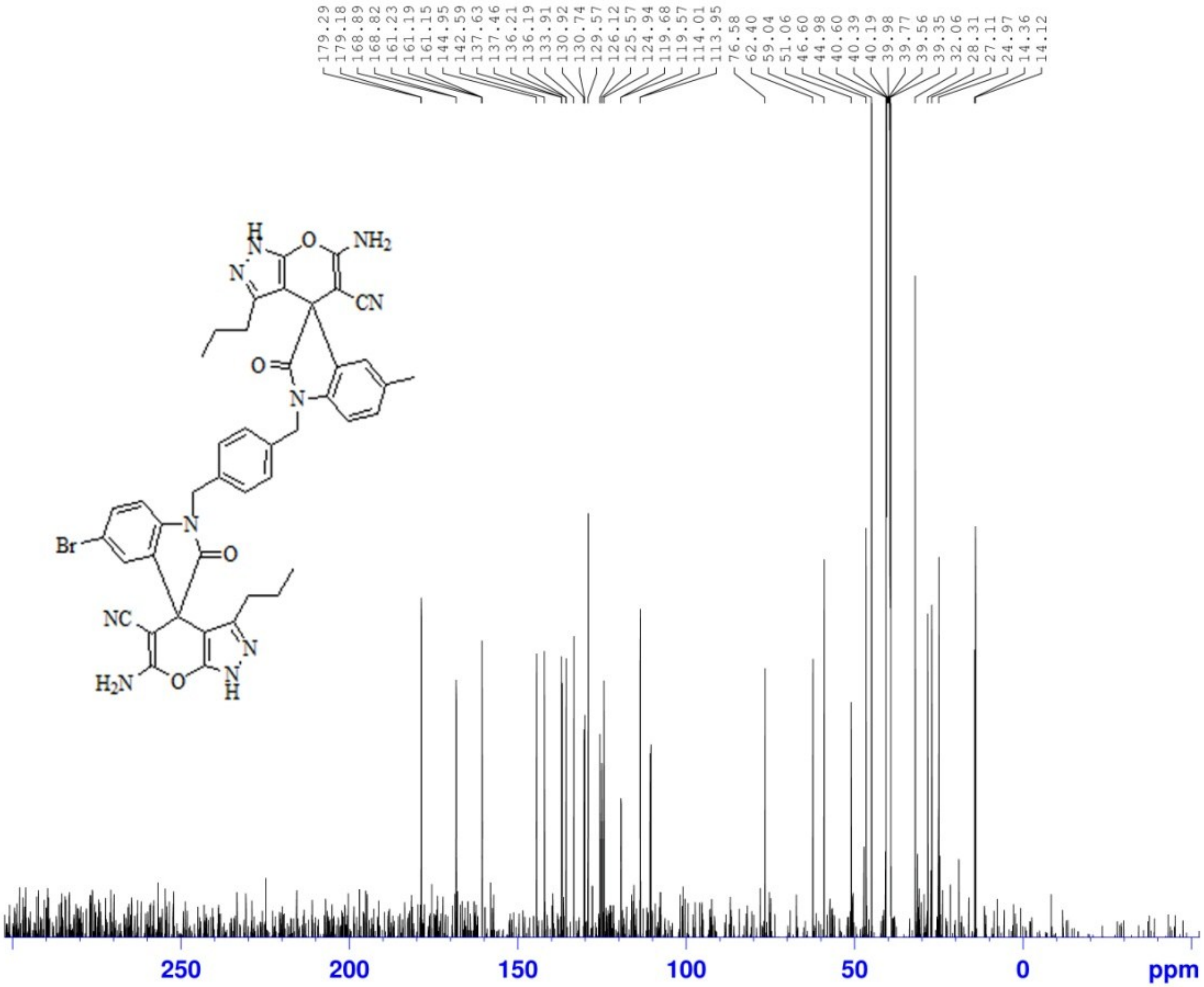

NAME	Khalij Fars UN
EXPNO	2458
PROCNO	1
Date_	20170722
Time	15.18
INSTRUM	spect
PROBHD	5 mm PABBO BB-
PULPROG	zgpg30
TD	65536
SOLVENT	DMSO
NS	4400
DS	0
SWH	35714.285 Hz
FIDRES	0.544957 Hz
AQ	0.9175540 sec
RG	2050
DW	14.000 usec
DE	6.50 usec
TE	295.1 K
D1	1.00000000 sec
D11	0.03000000 sec
TD0	1

======== CHANNEL f1 ========

NUC1	13C
P1	9.00 usec
PL1	-0.90 dB
PL1W	42.02801895 W
SFO1	100.6479784 MHz

======== CHANNEL f2 ========

CPDPRG2	waltz16
NUC2	1H
PCPD2	90.00 usec
PL2	-2.00 dB
PL12	14.48 dB
PL13	17.90 dB
PL2W	11.86359406 W
PL12W	0.26681873 W
PL13W	0.12139934 W
SFO2	400.2216009 MHz
SI	32768
SF	100.6353990 MHz
WDW	EM
SSB	0
LB	1.00 Hz
GB	0
PC	1.40

methyl 2'-amino-1-(6-(2'-amino-3'-(methoxycarbonyl)-5,7'-dimethyl-2,5'-dioxo-5'H-spiro[indoline-3,4'-pyrano[4,3-b]pyran]-1-yl)hexyl)-5-bromo-7'-methyl-2,5'-dioxo-5'H-spiro[indoline-3,4'-pyrano[4,3-b]pyran]-3'-carboxylate **6f**

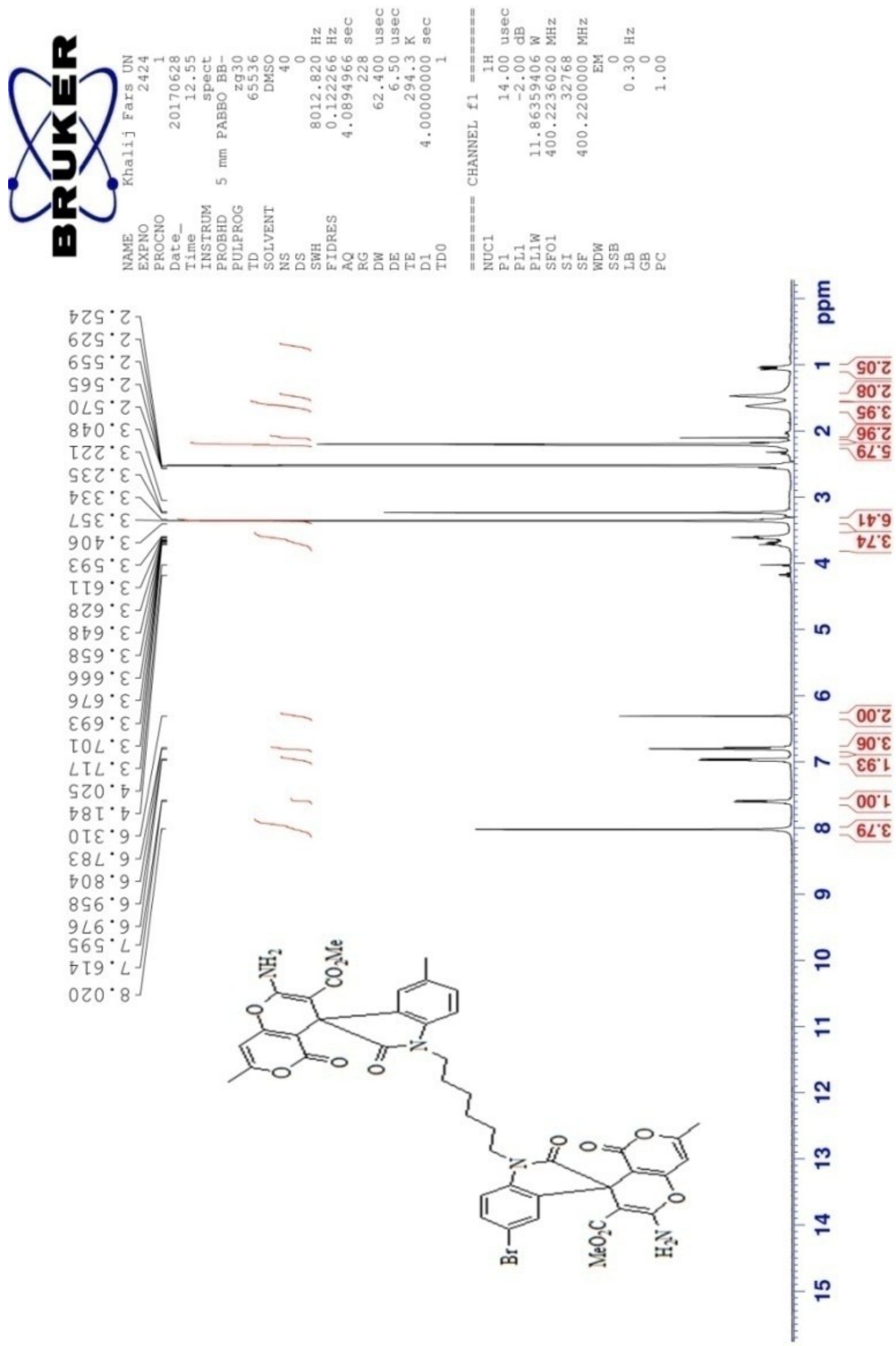

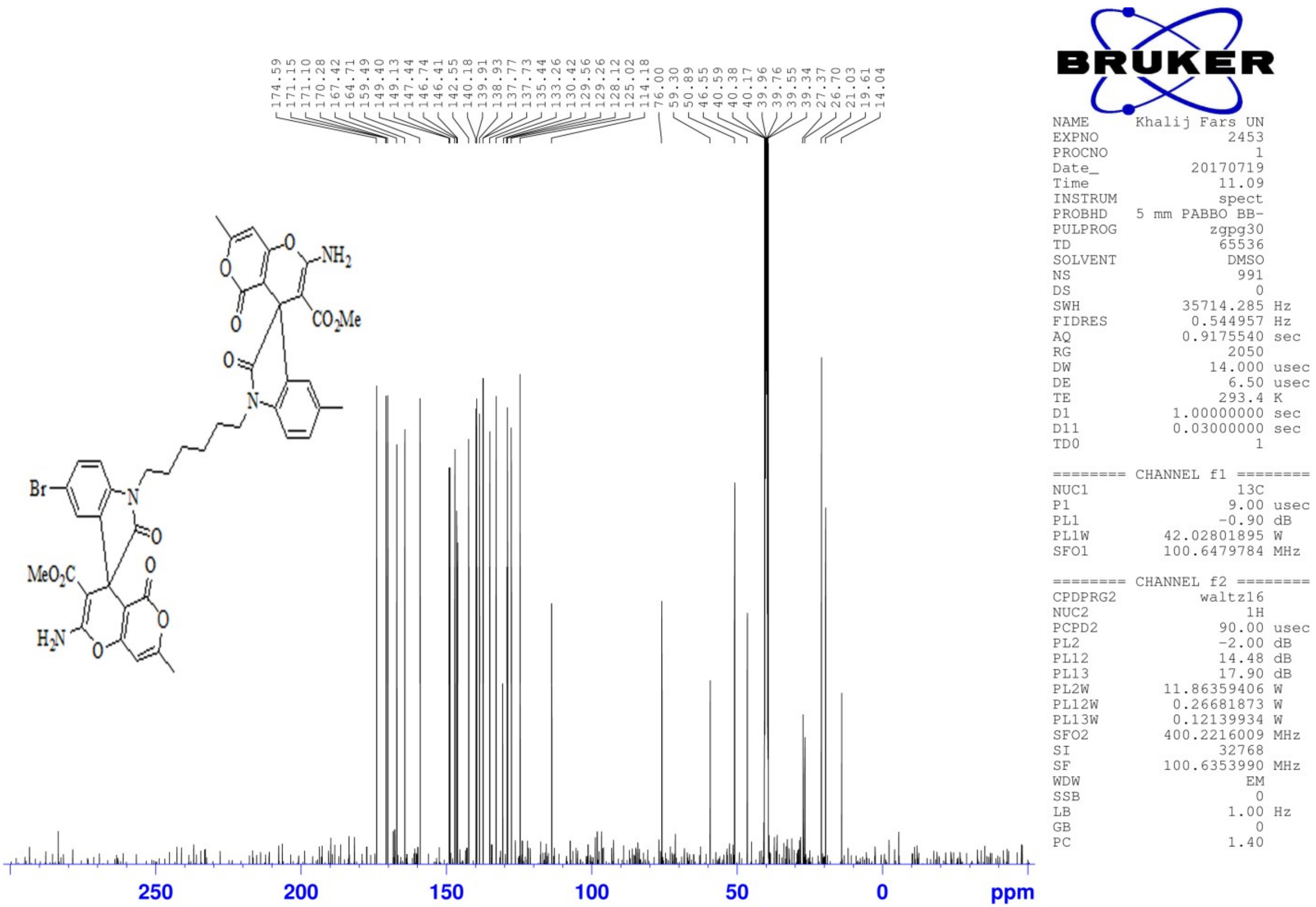
174.59
171.15
171.10
170.28
167.42
164.71
159.49
149.40
149.13
147.44
146.74
146.41
142.55
140.18
139.91
138.93
137.77
137.73
135.44
133.26
130.42
129.56
129.26
128.12
125.02
114.18
76.00
59.30
50.89
46.55
40.59
40.38
40.17
39.96
39.76
39.55
39.34
27.37
26.70
21.03
19.61
14.04
NH2
CO2Me
Br
N
MeO2C
H2N
250
200
150
100
50
0
ppm
NAME Khalij Fars UN
EXPNO 2453
PROCNO 1
Date_ 20170719
Time 11.09
INSTRUM spect
PROBHD 5 mm PABBO BB-
PULPROG zgpg30
TD 65536
SOLVENT DMSO
NS 991
DS 0
SWH 35714.285 Hz
FIDRES 0.544957 Hz
AQ 0.9175540 sec
RG 2050
DW 14.000 usec
DE 6.50 usec
TE 293.4 K
D1 1.00000000 sec
D11 0.03000000 sec
TD0 1
======== CHANNEL f1 ========
NUC1 13C
P1 9.00 usec
PL1 -0.90 dB
PL1W 42.02801895 W
SFO1 100.6479784 MHz
======== CHANNEL f2 ========
CPDPRG2 waltz16
NUC2 1H
PCPD2 90.00 usec
PL2 -2.00 dB
PL12 14.48 dB
PL13 17.90 dB
PL2W 11.86359406 W
PL12W 0.26681873 W
PL13W 0.12139934 W
SFO2 400.2216009 MHz
SI 32768
SF 100.6353990 MHz
WDW EM
SSB 0
LB 1.00 Hz
GB 0
PC 1.40

2'-amino-1-(4-(2'-amino-3'-cyano-5,7'-dimethyl-2,5'-dioxo-5'H-spiro[indoline-3,4'-pyrano[4,3-b]pyran]-1-yl)butyl)-7'-methyl-2,5'-dioxo-5'H-spiro[indoline-3,4'-pyrano[4,3-b]pyran]-3'-carbonitrile **6g**

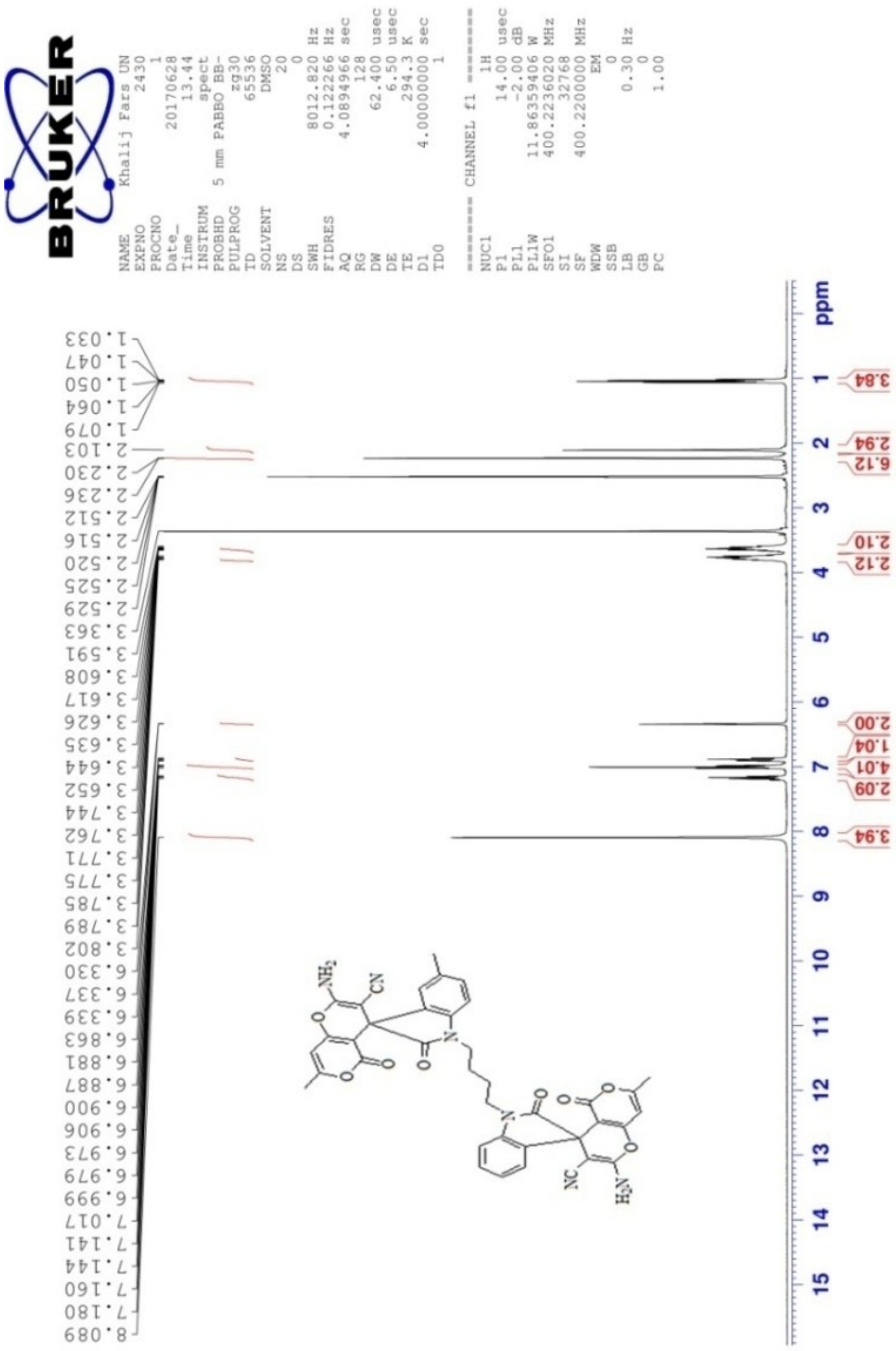

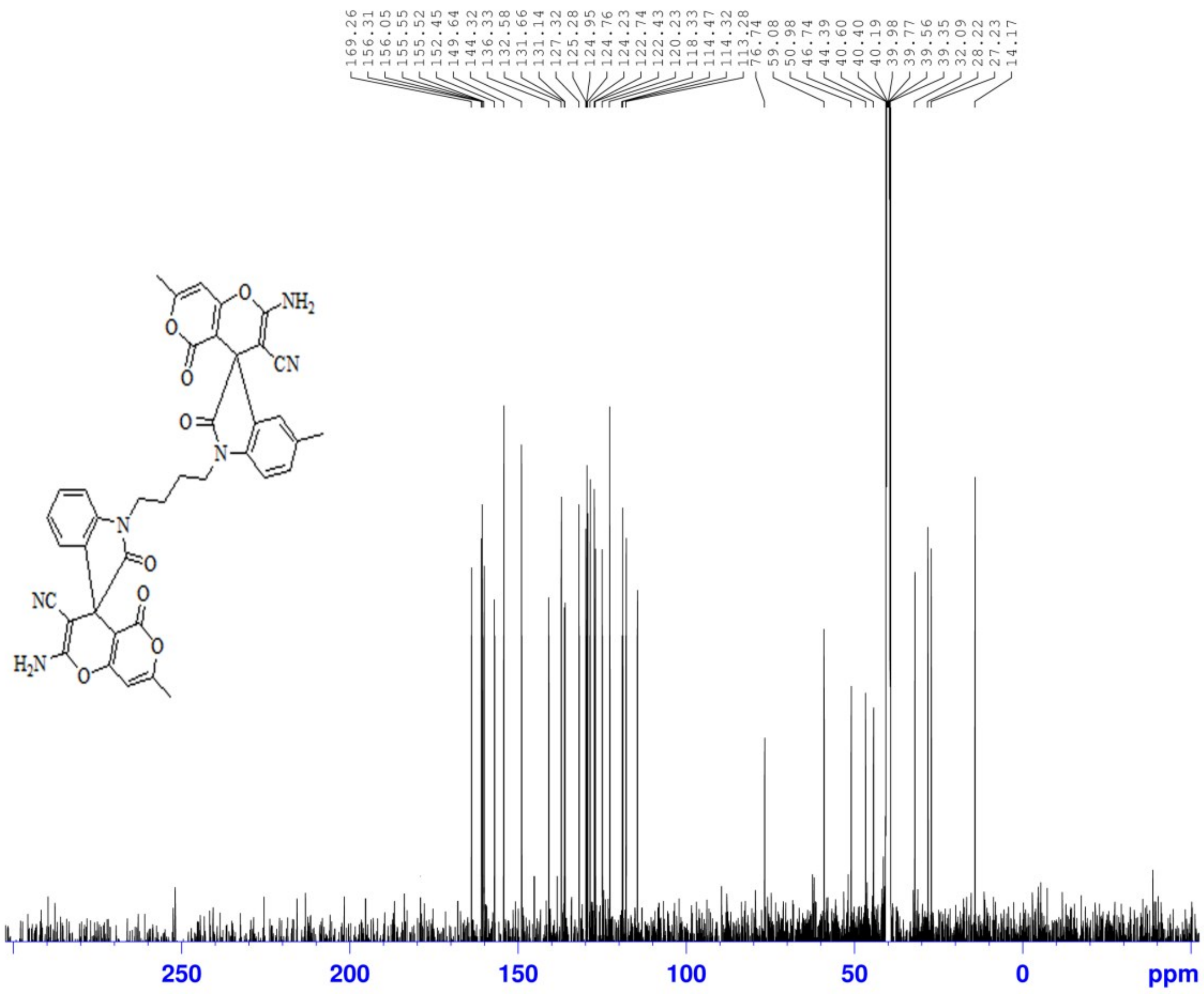

NAME	Khalij Fars UN	
EXPNO	2448	
PROCNO	1	
Date_	20170718	
Time	21.19	
INSTRUM	spect	
PROBHD	5 mm PABBO BB-	
PULPROG	zgpg30	
TD	65536	
SOLVENT	DMSO	
NS	2836	
DS	0	
SWH	35714.285	Hz
FIDRES	0.544957	Hz
AQ	0.9175540	sec
RG	2050	
DW	14.000	usec
DE	6.50	usec
TE	294.0	K
D1	1.00000000	sec
D11	0.03000000	sec
TD0	1	
======== CHANNEL f1 ========		
NUC1	13C	
P1	9.00	usec
PL1	-0.90	dB
PL1W	42.02801895	W
SFO1	100.6479784	MHz
======== CHANNEL f2 ========		
CPDPRG2	waltz16	
NUC2	1H	
PCPD2	90.00	usec
PL2	-2.00	dB
PL12	14.48	dB
PL13	17.90	dB
PL2W	11.86359406	W
PL12W	0.26681873	W
PL13W	0.12139934	W
SFO2	400.2216009	MHz
SI	32768	
SF	100.6353990	MHz
WDW	EM	
SSB	0	
LB	1.00	Hz
GB	0	
PC	1.40	

ethyl 2-amino-1'-(4-((2-amino-3-(ethoxycarbonyl)-5'-methyl-2',5-dioxo-5,6,7,8-tetrahydrospiro[chromene-4,3'-indolin]-1'-yl)methyl)benzyl)-5'-bromo-2',5-dioxo-5,6,7,8-tetrahydrospiro[chromene-4,3'-indoline]-3-carboxylate **6h**

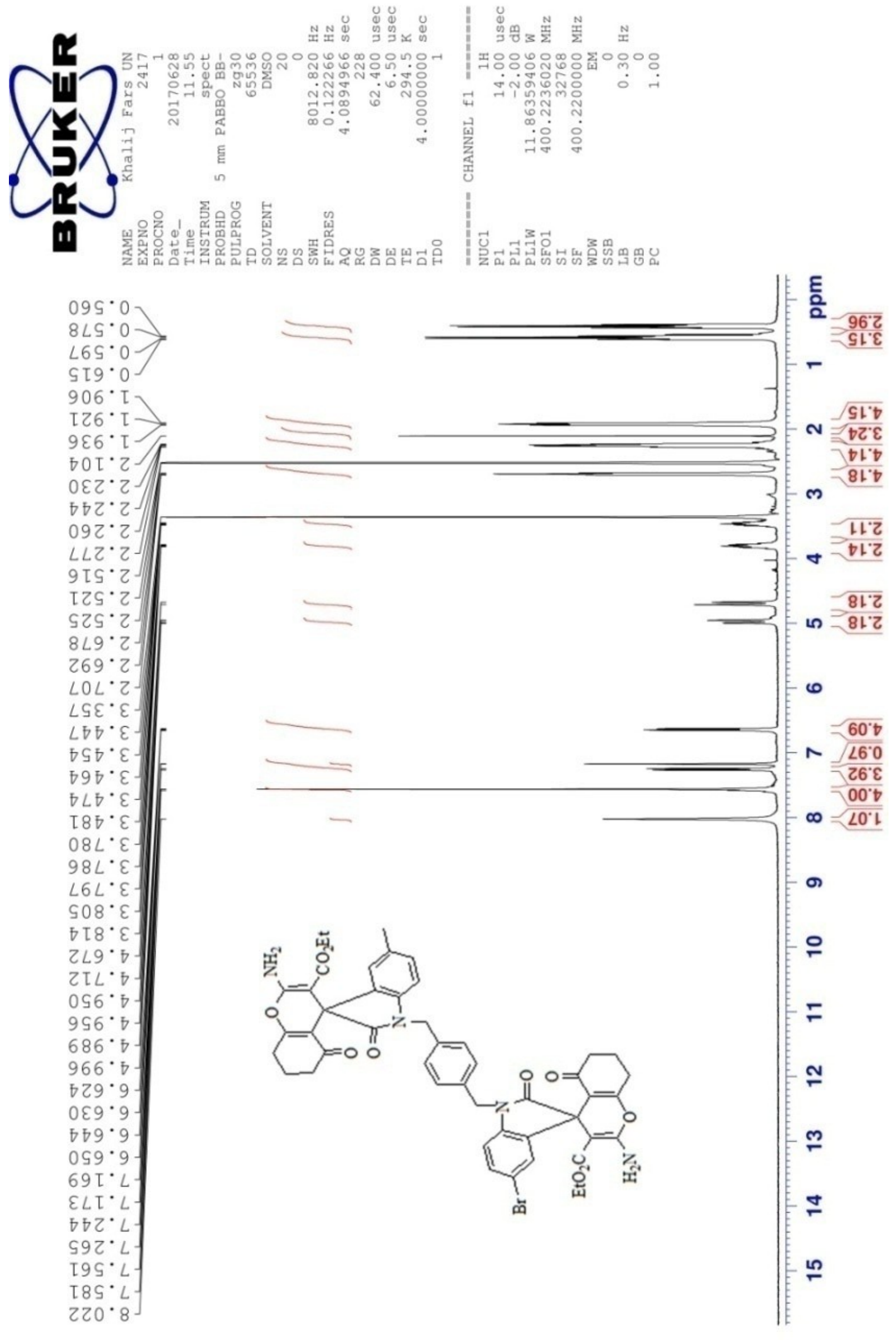

Sample solubility was too low for 13C-NMR
even after heating.

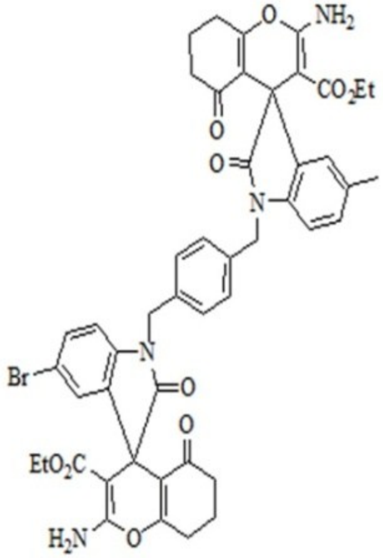

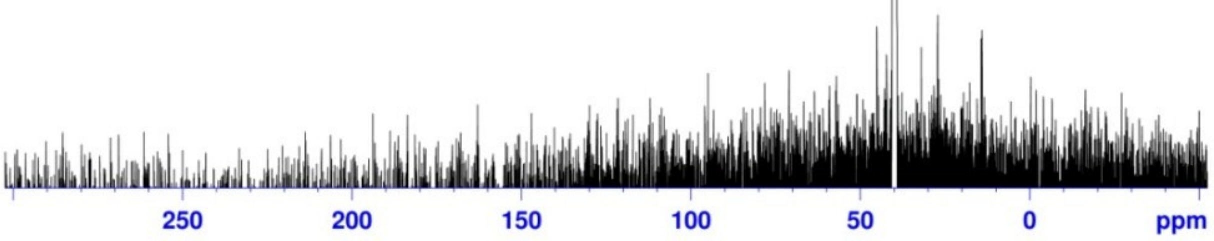

BRUKER

NAME	Khalij Fars UN
EXPNO	2455
PROCNO	1
Date_	20170719
Time	13.51
INSTRUM	spect
PROBHD	5 mm PABBO BB-
PULPROG	zgpg30
TD	65536
SOLVENT	DMSO
NS	1598
DS	0
SWH	35714.285 Hz
FIDRES	0.544957 Hz
AQ	0.9175540 sec
RG	2050
DW	14.000 usec
DE	6.50 usec
TE	293.8 K
D1	1.00000000 sec
D11	0.03000000 sec
TD0	1
======== CHANNEL f1 ========	
NUC1	13C
P1	9.00 usec
PL1	-0.90 dB
PL1W	42.02801895 W
SFO1	100.6479784 MHz
======== CHANNEL f2 ========	
CPDPRG2	waltz16
NUC2	1H
PCPD2	90.00 usec
PL2	-2.00 dB
PL12	14.48 dB
PL13	17.90 dB
PL2W	11.86359406 W
PL12W	0.26681873 W
PL13W	0.12139934 W
SFO2	400.2216009 MHz
SI	32768
SF	100.6353990 MHz
WDW	EM
SSB	0
LB	1.00 Hz
GB	0
PC	1.40

2-amino-1'-(4-((2-amino-3-cyano-5',7,7-trimethyl-2',5-dioxo-5,6,7,8-tetrahydrospiro[chromene-4,3'-indolin]-1'-yl)methyl)benzyl)-5'-chloro-7,7-dimethyl-2',5-dioxo-5,6,7,8-tetrahydrospiro[chromene-4,3'-indoline]-3-carbonitrile **6**i

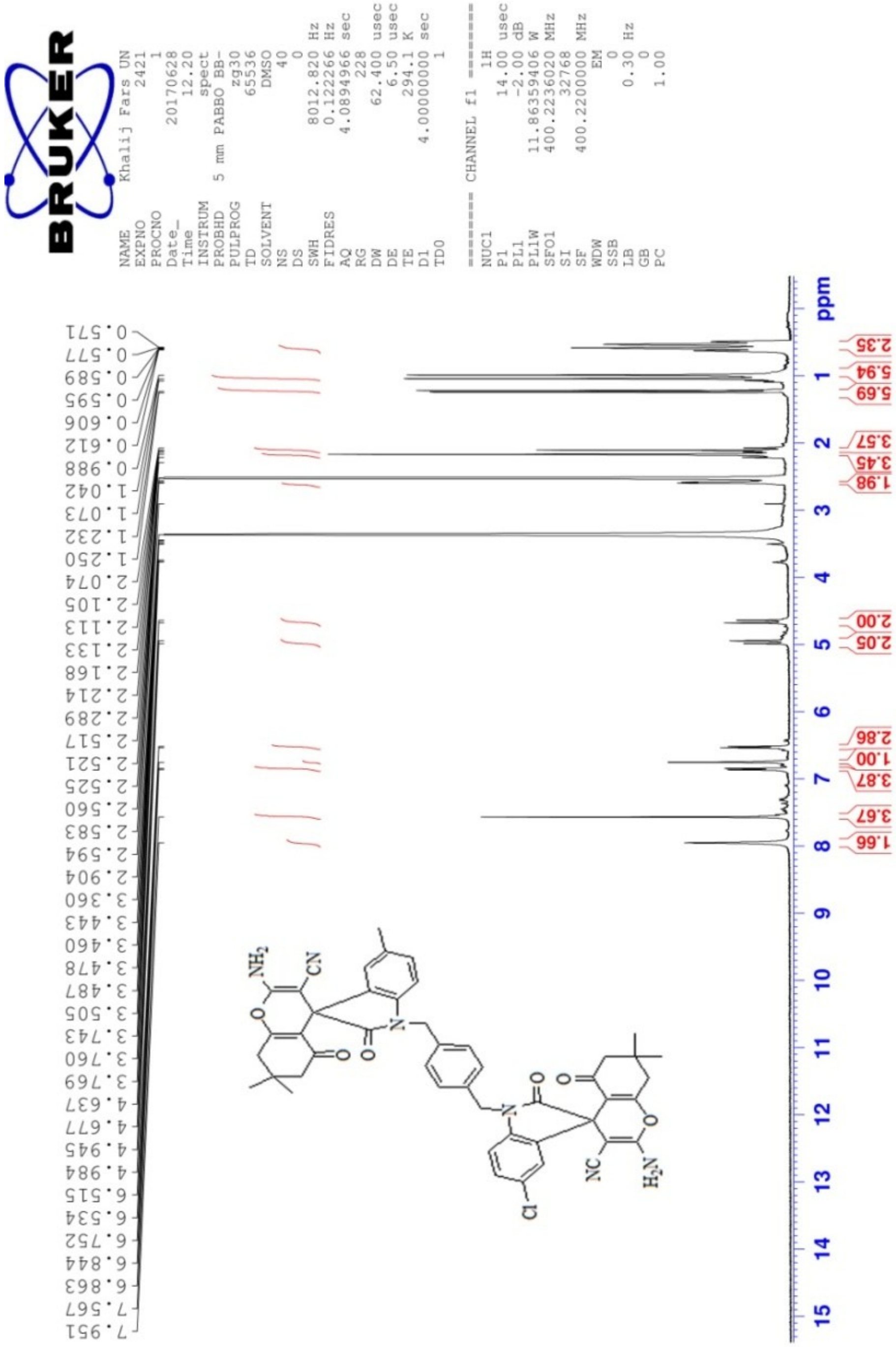

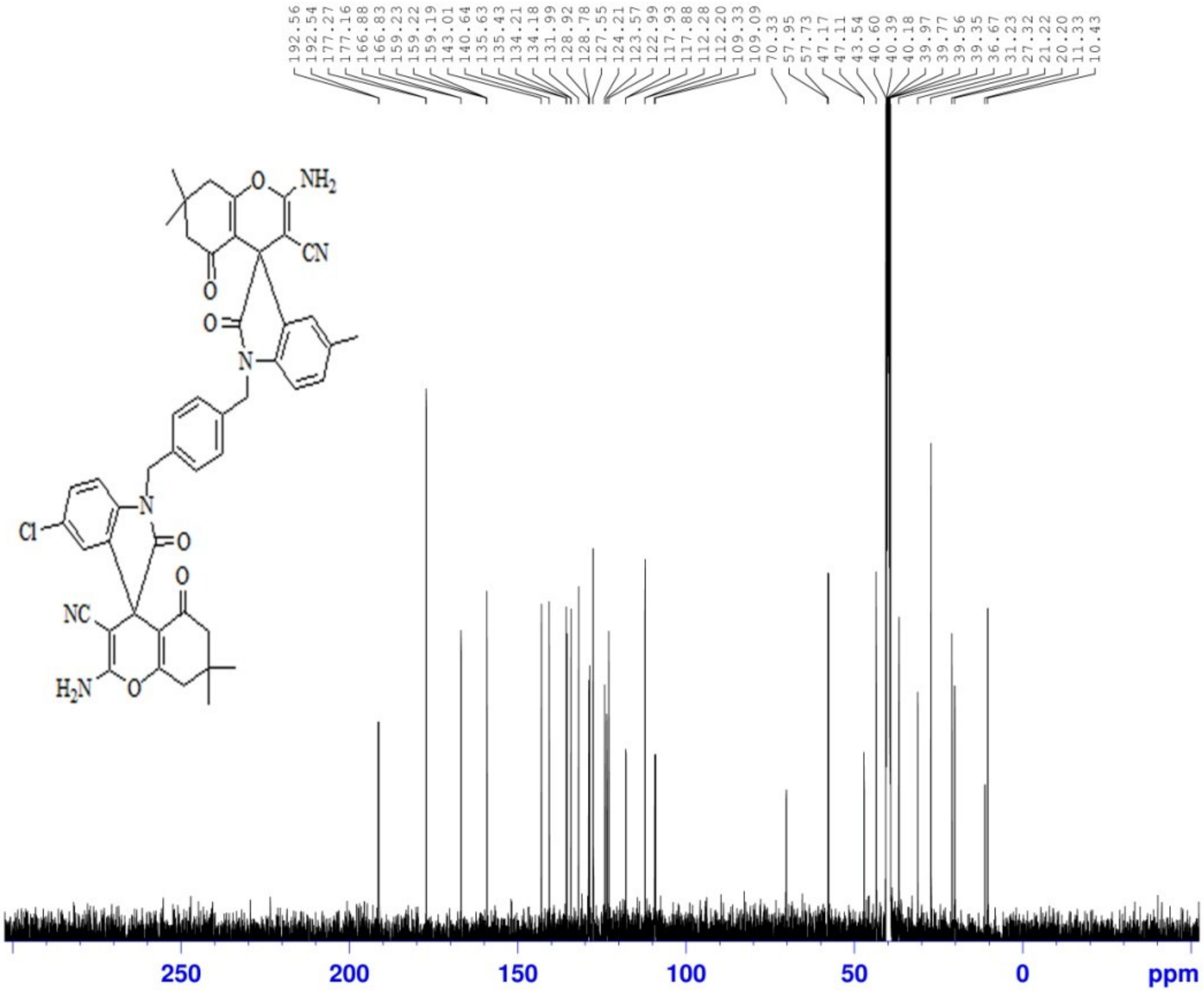

NAME Khalij Fars UN
EXPNO 2446
PROCNO 1
Date_ 20170718
Time 17.34
INSTRUM spect
PROBHD 5 mm PABBO BB-
PULPROG zgpg30
TD 65536
SOLVENT DMSO
NS 708
DS 0
SWH 35714.285 Hz
FIDRES 0.544957 Hz
AQ 0.9175540 sec
RG 2050
DW 14.000 usec
DE 6.50 usec
TE 294.1 K
D1 1.00000000 sec
D11 0.03000000 sec
TD0 1

======== CHANNEL f1 ========
NUC1 13C
P1 9.00 usec
PL1 -0.90 dB
PL1W 42.02801895 W
SFO1 100.6479784 MHz

======== CHANNEL f2 ========
CPDPRG2 waltz16
NUC2 1H
PCPD2 90.00 usec
PL2 -2.00 dB
PL12 14.48 dB
PL13 17.90 dB
PL2W 11.86359406 W
PL12W 0.26681873 W
PL13W 0.12139934 W
SFO2 400.2216009 MHz
SI 32768
SF 100.6353990 MHz
WDW EM
SSB 0
LB 1.00 Hz
GB 0
PC 1.40

ethyl 2-amino-1'-(4-((2-amino-3-(ethoxycarbonyl)-5',7,7-trimethyl-2',5-dioxo-5,6,7,8-tetrahydrospiro[chromene-4,3'-indolin]-1'-yl)methyl)benzyl)-5'-bromo-7,7-dimethyl-2',5-dioxo-5,6,7,8-tetrahydrospiro[chromene-4,3'-indoline]-3-carboxylate **6j**

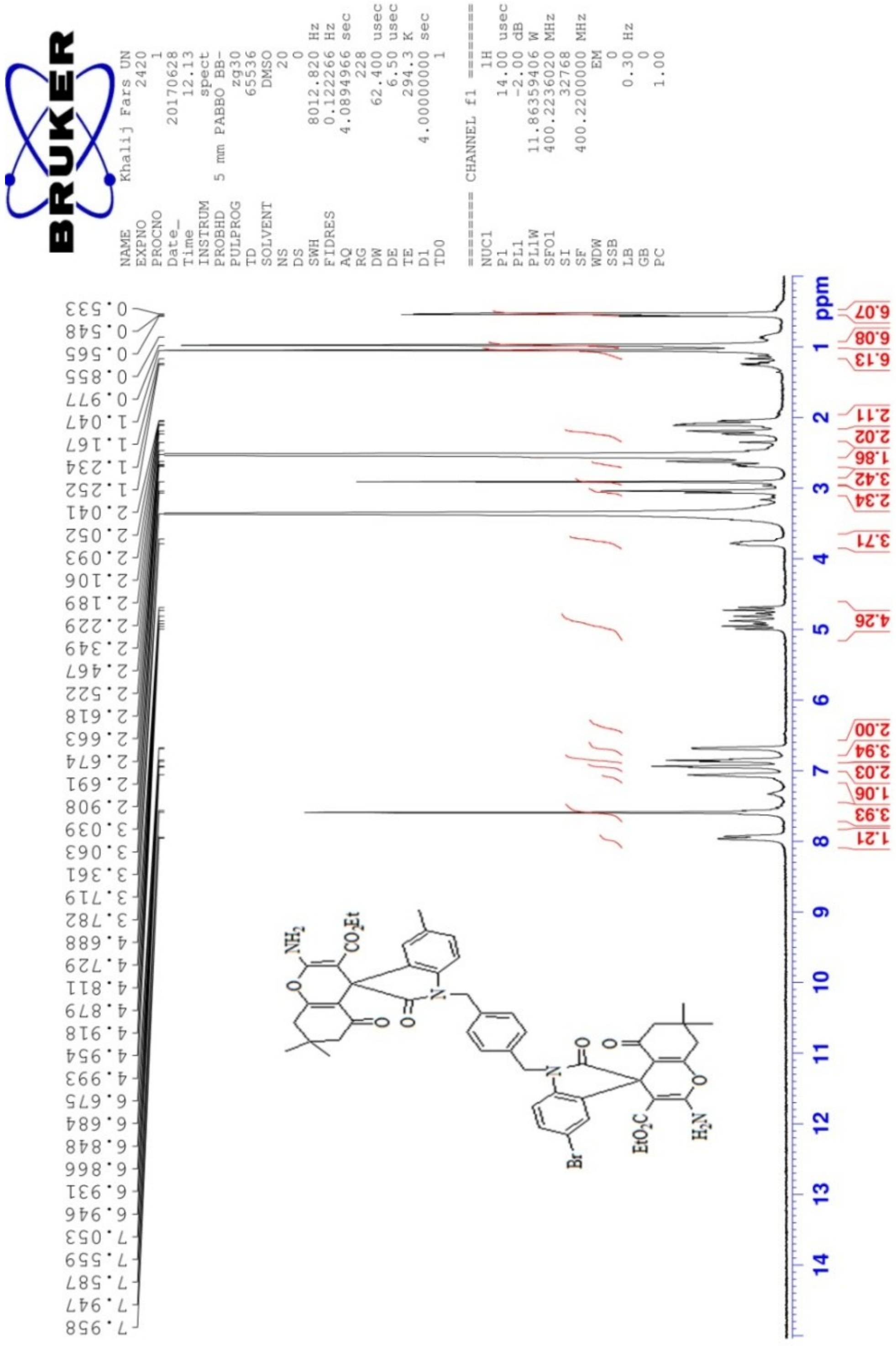

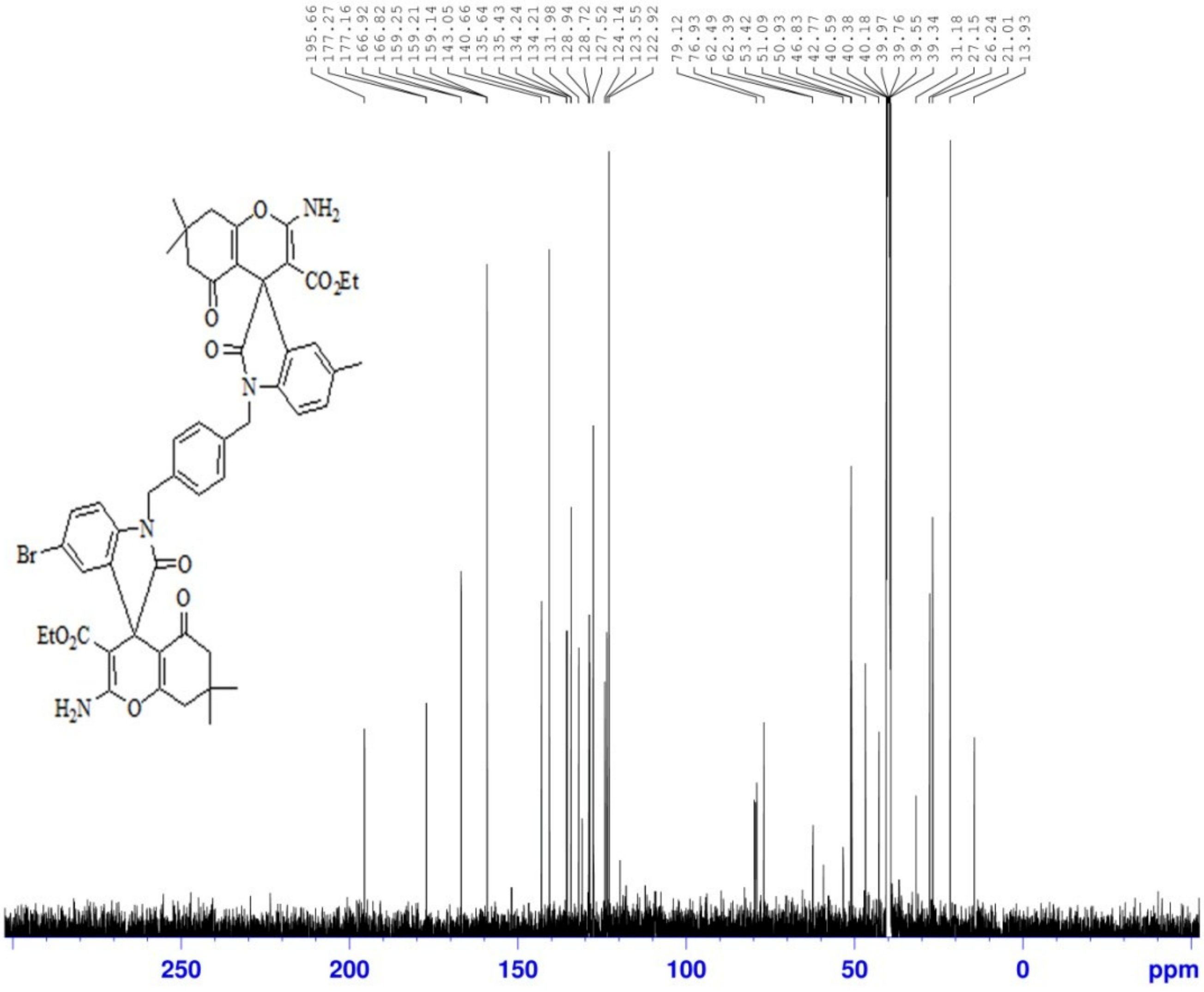

NAME	Khalij Fars UN	
EXPNO	2451	
PROCNO	1	
Date_	20170719	
Time	8.33	
INSTRUM	spect	
PROBHD	5 mm PABBO BB-	
PULPROG	zgpg30	
TD	65536	
SOLVENT	DMSO	
NS	1302	
DS	0	
SWH	35714.285	Hz
FIDRES	0.544957	Hz
AQ	0.9175540	sec
RG	2050	
DW	14.000	usec
DE	6.50	usec
TE	293.1	K
D1	1.00000000	sec
D11	0.03000000	sec
TD0	1	

======== CHANNEL f1 ========

NUC1	13C	
P1	9.00	usec
PL1	-0.90	dB
PL1W	42.02801895	W
SFO1	100.6479784	MHz

======== CHANNEL f2 ========

CPDPRG2	waltz16	
NUC2	1H	
PCPD2	90.00	usec
PL2	-2.00	dB
PL12	14.48	dB
PL13	17.90	dB
PL2W	11.86359406	W
PL12W	0.26681873	W
PL13W	0.12139934	W
SFO2	400.2216009	MHz
SI	32768	
SF	100.6353990	MHz
WDW	EM	
SSB	0	
LB	1.00	Hz
GB	0	
PC	1.40	

methyl 2'-amino-1-(4-(2'-amino-3'-(methoxycarbonyl)-5-methyl-2,5'-dioxo-5'H-spiro[indoline-3,4'-pyrano[3,2-c]chromen]-1-yl)butyl)-5-bromo-2,5'-dioxo-5'H-spiro[indoline-3,4'-pyrano[3,2-c]chromene]-3'-carboxylate **6k**

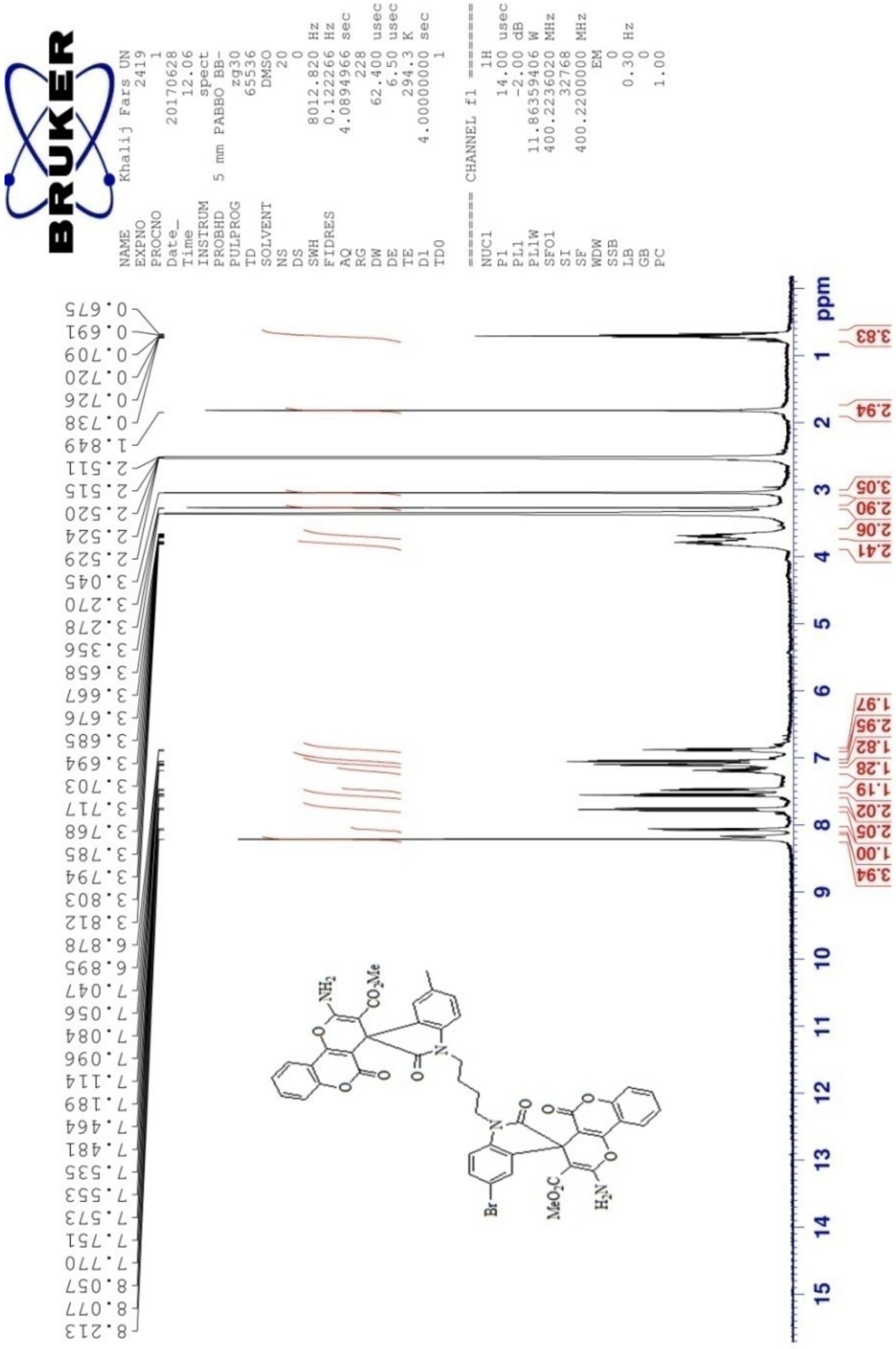

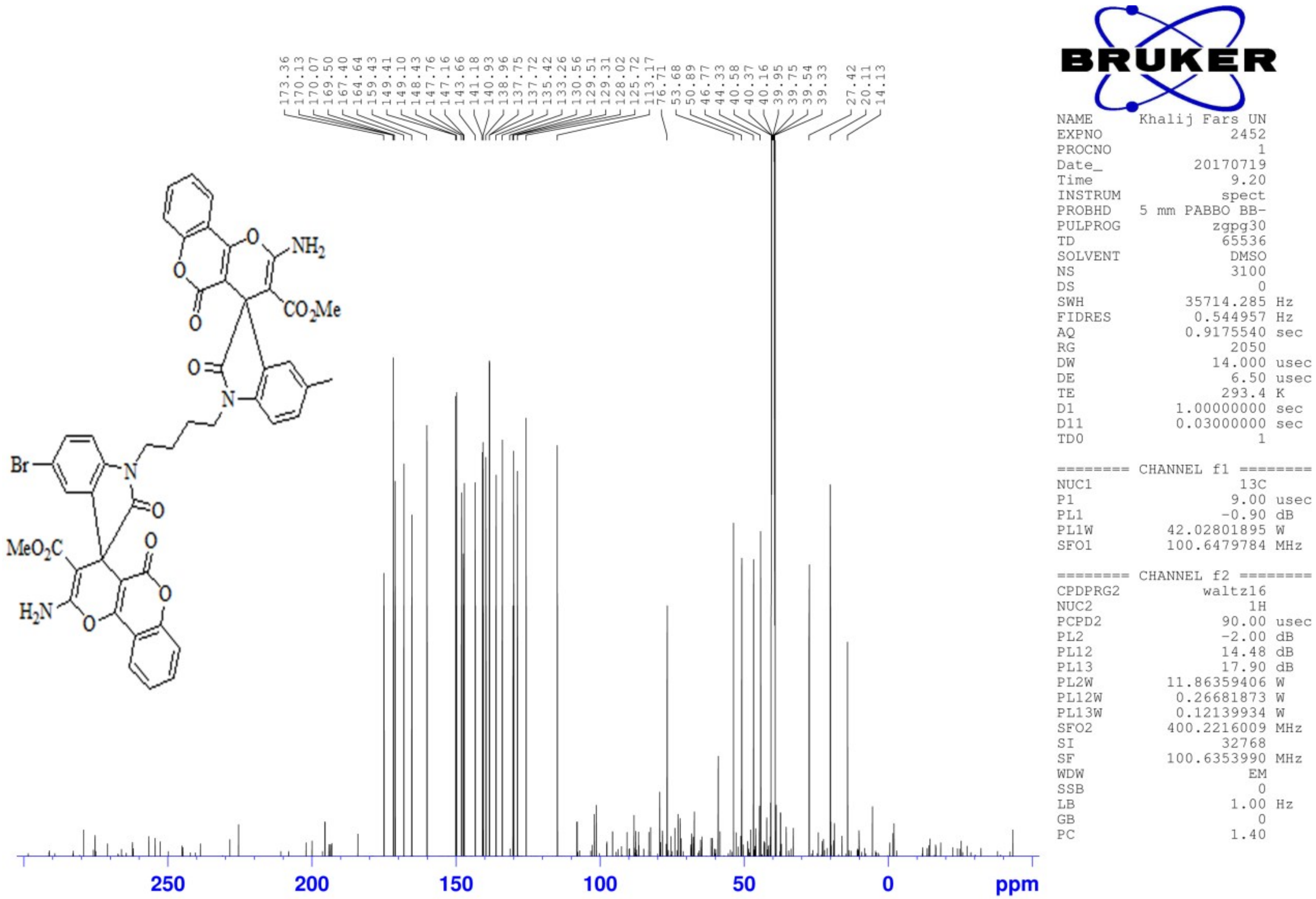

173.36
170.13
170.07
169.50
167.40
164.64
159.43
149.41
149.10
148.43
147.76
147.16
143.66
141.18
140.93
138.96
137.75
137.72
135.42
133.26
130.56
129.51
129.31
128.02
125.72
113.17
76.71
53.68
50.89
46.77
44.33
40.58
40.37
40.16
39.95
39.75
39.54
39.33
27.42
20.11
14.13
NH2
CO2Me
O
N
Br
MeO2C
H2N
250
200
150
100
50
0
ppm
BRUKER
NAME Khalij Fars UN
EXPNO 2452
PROCNO 1
Date_ 20170719
Time 9.20
INSTRUM spect
PROBHD 5 mm PABBO BB-
PULPROG zgpg30
TD 65536
SOLVENT DMSO
NS 3100
DS 0
SWH 35714.285 Hz
FIDRES 0.544957 Hz
AQ 0.9175540 sec
RG 2050
DW 14.000 usec
DE 6.50 usec
TE 293.4 K
D1 1.00000000 sec
D11 0.03000000 sec
TD0 1
======== CHANNEL f1 ========
NUC1 13C
P1 9.00 usec
PL1 -0.90 dB
PL1W 42.02801895 W
SFO1 100.6479784 MHz
======== CHANNEL f2 ========
CPDPRG2 waltz16
NUC2 1H
PCPD2 90.00 usec
PL2 -2.00 dB
PL12 14.48 dB
PL13 17.90 dB
PL2W 11.86359406 W
PL12W 0.26681873 W
PL13W 0.12139934 W
SFO2 400.2216009 MHz
SI 32768
SF 100.6353990 MHz
WDW EM
SSB 0
LB 1.00 Hz
GB 0
PC 1.40

ethyl 6'-amino-1-(4-(6'-amino-5'-(ethoxycarbonyl)-3',5-dimethyl-2-oxo-1'H-spiro[indoline-3,4'-pyrano[2,3-c]pyrazol]-1-yl)butyl)-3'-methyl-2-oxo-1'H-spiro[indoline-3,4'-pyrano[2,3-c]pyrazole]-5'-carboxylate **6l**

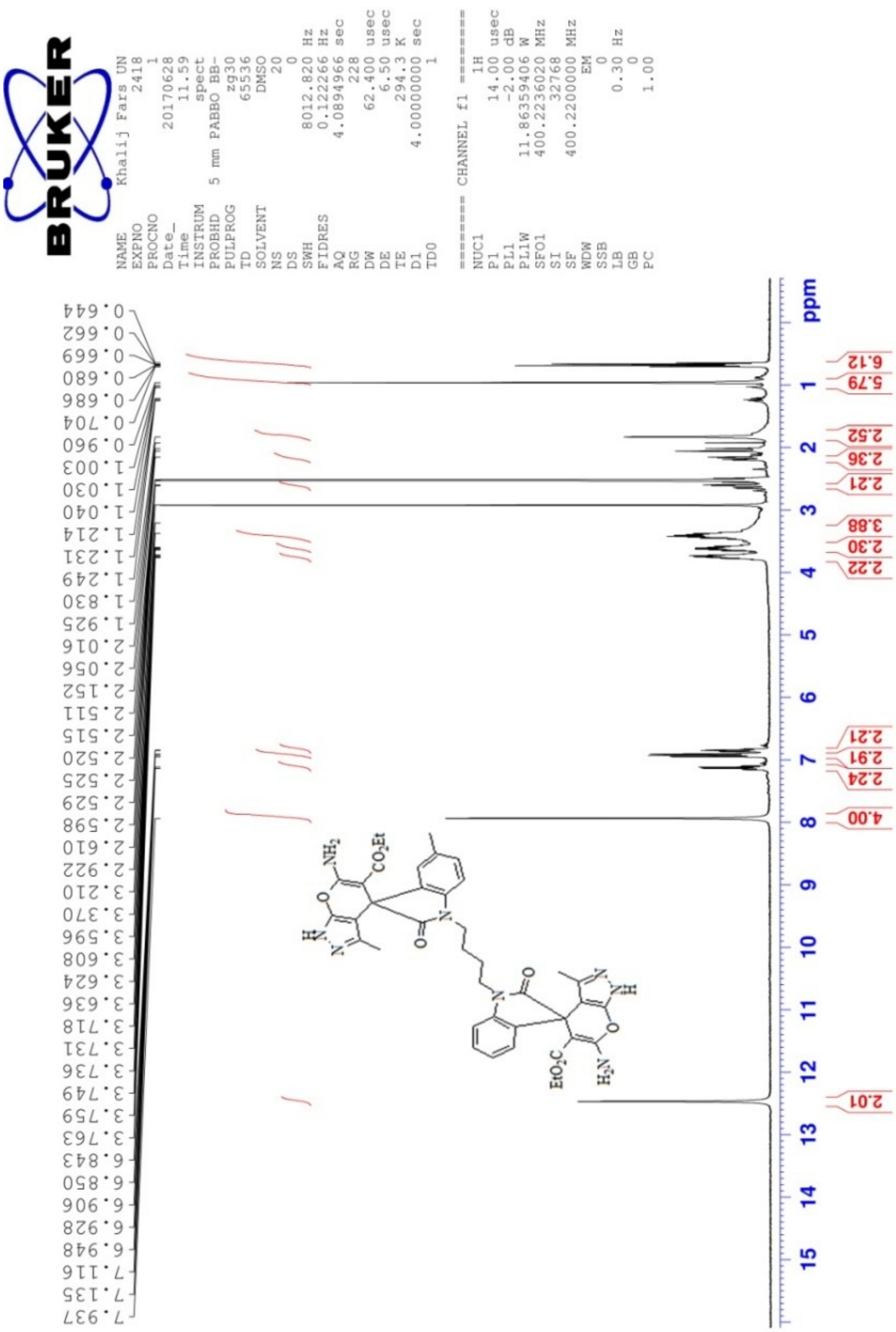

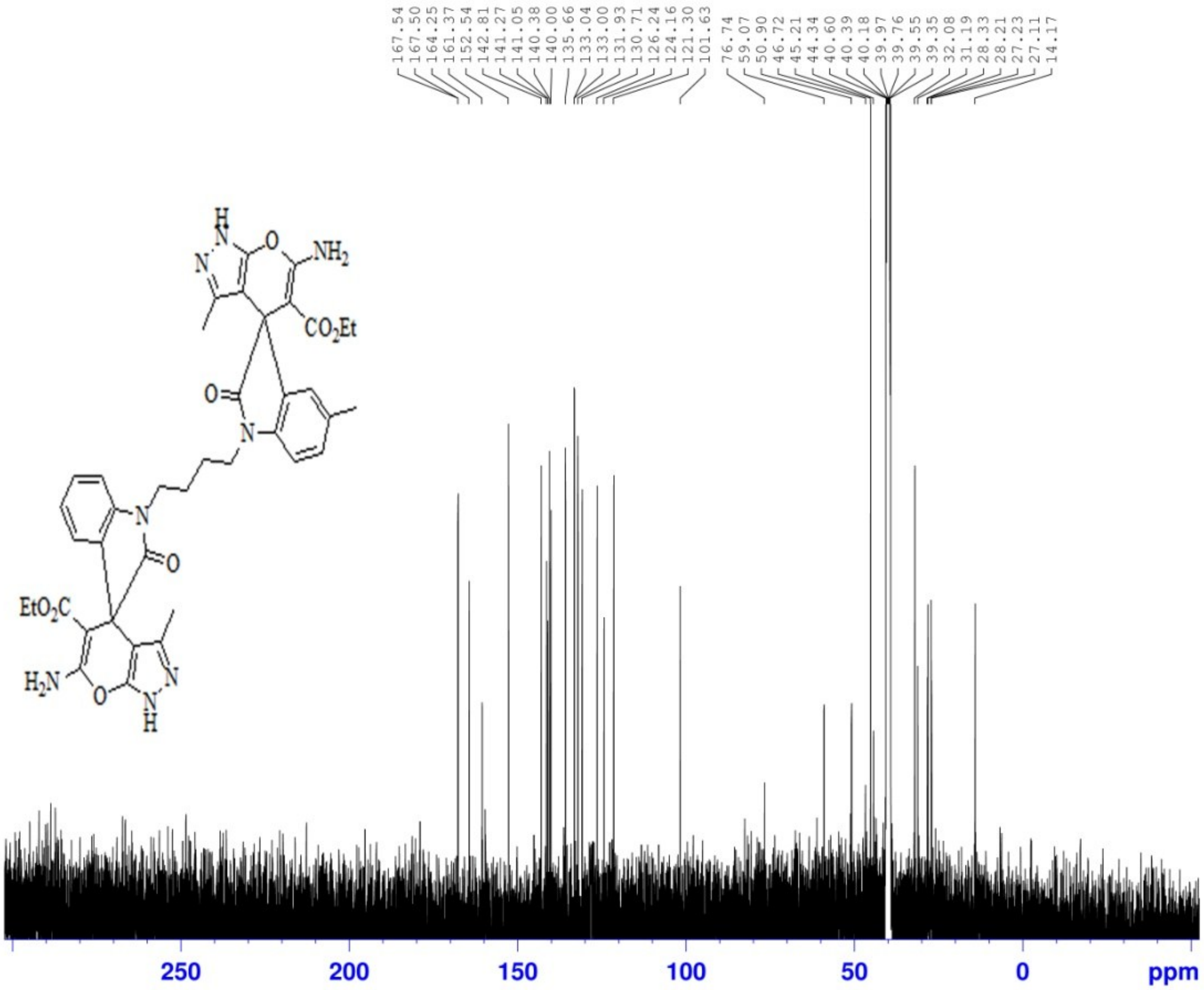

NAME	Khalij Fars UN
EXPNO	2449
PROCNO	1
Date_	20170719
Time	2.31
INSTRUM	spect
PROBHD	5 mm PABBO BB-
PULPROG	zgpg30
TD	65536
SOLVENT	DMSO
NS	8192
DS	0
SWH	35714.285 Hz
FIDRES	0.544957 Hz
AQ	0.9175540 sec
RG	2050
DW	14.000 usec
DE	6.50 usec
TE	293.8 K
D1	1.00000000 sec
D11	0.03000000 sec
TD0	1
======== CHANNEL f1 ========	
NUC1	13C
P1	9.00 usec
PL1	-0.90 dB
PL1W	42.02801895 W
SFO1	100.6479784 MHz
======== CHANNEL f2 ========	
CPDPRG2	waltz16
NUC2	1H
PCPD2	90.00 usec
PL2	-2.00 dB
PL12	14.48 dB
PL13	17.90 dB
PL2W	11.86359406 W
PL12W	0.26681873 W
PL13W	0.12139934 W
SFO2	400.2216009 MHz
SI	32768
SF	100.6353990 MHz
WDW	EM
SSB	0
LB	1.00 Hz
GB	0
PC	1.40

methyl 2-amino-1'-(6-(2-amino-3-(methoxycarbonyl)-5',7,7-trimethyl-2',5-dioxo-5,6,7,8-tetrahydrospiro[chromene-4,3'-indolin]-1'-yl)hexyl)-7,7-dimethyl-2',5-dioxo-5,6,7,8-tetrahydrospiro[chromene-4,3'-indoline]-3-carboxylate **6m**

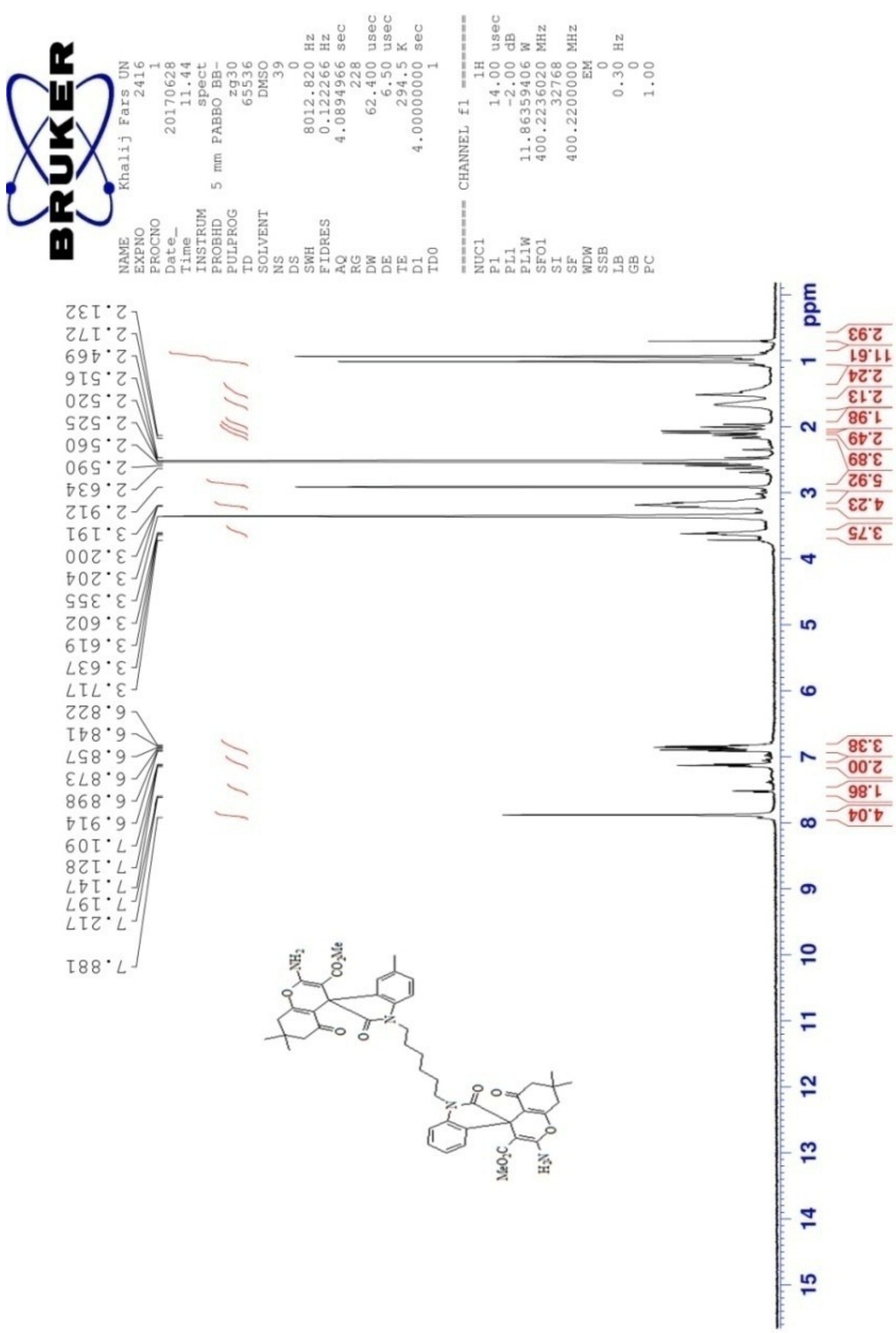

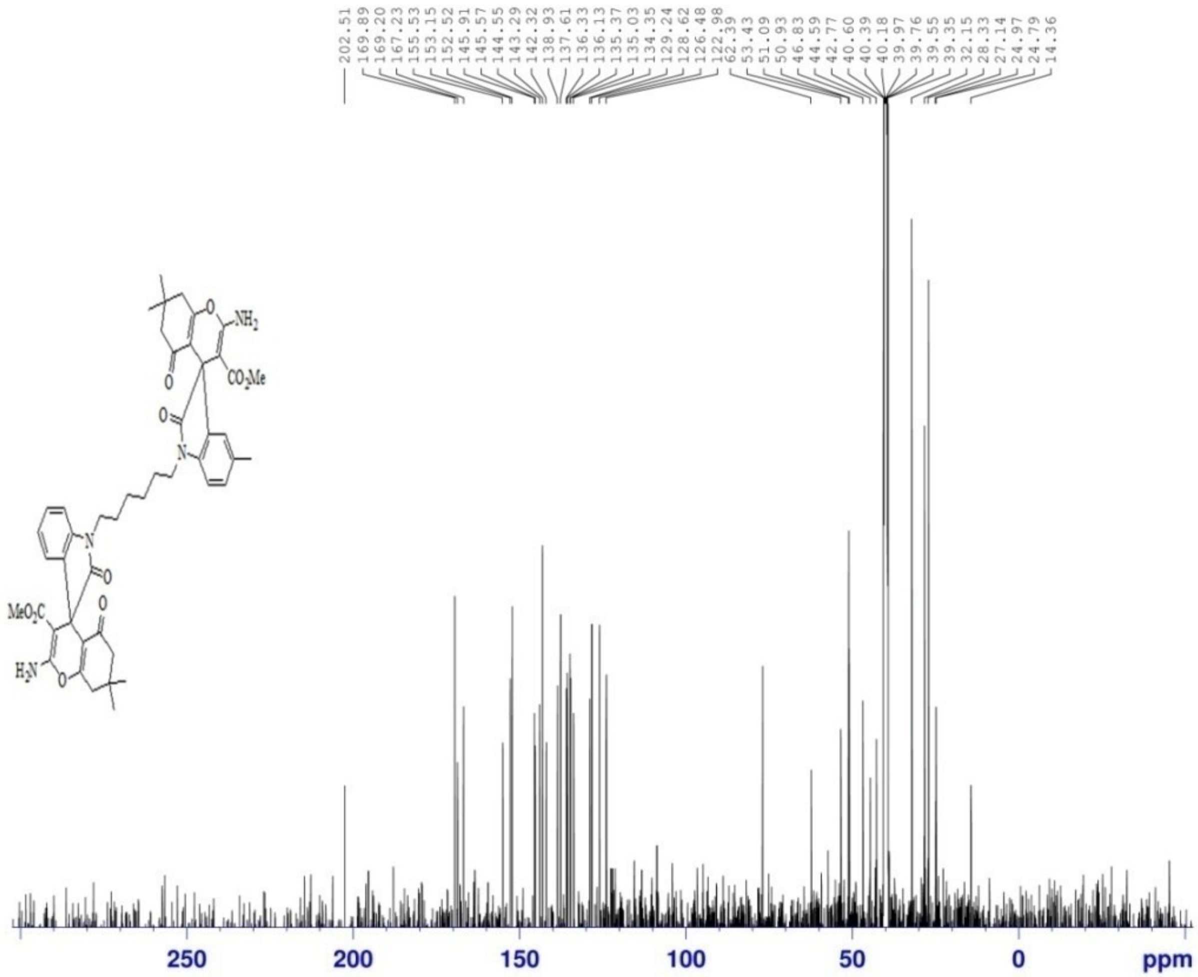

NAME	Khalij Fars UN	
EXPNO	2454	
PROCNO	1	
Date_	20170719	
Time	11.46	
INSTRUM	spect	
PROBHD	5 mm PABBO BB-	
PULPROG	zgpg30	
TD	65536	
SOLVENT	DMSO	
NS	3624	
DS	0	
SWH	35714.285	Hz
FIDRES	0.544957	Hz
AQ	0.9175540	sec
RG	2050	
DW	14.000	usec
DE	6.50	usec
TE	293.5	K
D1	1.00000000	sec
D11	0.03000000	sec
TD0	1	
======== CHANNEL f1 ========		
NUC1	13C	
P1	9.00	usec
PL1	-0.90	dB
PL1W	42.02801895	W
SFO1	100.6479784	MHz
======== CHANNEL f2 ========		
CPDPRG2	waltz16	
NUC2	1H	
PCPD2	90.00	usec
PL2	-2.00	dB
PL12	14.48	dB
PL13	17.90	dB
PL2W	11.86359406	W
PL12W	0.26681873	W
PL13W	0.12139934	W
SFO2	400.2216009	MHz
SI	32768	
SF	100.6353990	MHz
WDW	EM	
SSB	0	
LB	1.00	Hz
GB	0	
PC	1.40	
